# A reproducible open-data framework for interpretable breast cancer diagnosis and cytopathology education

**DOI:** 10.3389/fonc.2026.1921082

**Published:** 2026-10-26

**Authors:** Min Li, Xinxin Zhang, Xiaosu Xie, Yu Shi, Xueyang Hu, Nana Huang, Zilu Wang

**Affiliations:** 1 Department of Oncology, The First Affiliated Hospital of Anhui Medical University, Hefei, Anhui, China; 2 Department of Nuclear Medicine, The First Affiliated Hospital of University of Science and Technology of China (USTC), Hefei, Anhui, China; 3 Department of Oncology, The 901 Hospital of the Joint Logistics Support Force of the People’s Liberation Army of China, Hefei, Anhui, China

**Keywords:** breast cancer, cytopathology, decision-curve analysis, machine learning, model calibration and confirmation, reproducible research data, medical education

## Abstract

**Introduction:**

Quantitative nuclear morphology links cytopathological assessment with interpretable diagnostic modeling and provides a practical foundation for teaching medical artificial intelligence. This study evaluated a reproducible framework that connects nuclear feature interpretation, malignant-versus-benign classification, and evidence-based assessment of diagnostic decisions.

**Methods:**

The Wisconsin Diagnostic Breast Cancer dataset included 569 fine-needle aspiration samples (212 malignant and 357 benign) represented by 30 image-derived nuclear features. Seven supervised classifiers were compared using nested stratified cross-validation with five outer folds and four inner folds. Assessment covered ROC-AUC, precision-recall performance, probability calibration, training-derived decision thresholds, exploratory decision-curve analysis, feature importance, and leakage-controlled feature reduction.

**Results:**

The radial basis function support vector machine achieved a ROC-AUC of 0.995, while the interpretable linear support vector machine (SVM) achieved 0.994, without a significant pairwise difference from the radial basis function SVM or logistic regression. For the linear SVM, fold-specific Youden thresholds yielded held-out accuracy of 0.981, sensitivity of 0.962, specificity of 0.992, F1-score of 0.974, and Matthews correlation coefficient of 0.959. Sensitivity-prioritized thresholding increased sensitivity to 0.991, with specificity of 0.866. The Brier score was 0.021. Nuclear texture, size, symmetry, and contour-related characteristics contributed prominently to model interpretation. A leakage-controlled 20-feature model retained a ROC-AUC of 0.996 and a precision-recall area under the curve of 0.994.

**Discussion:**

The framework integrates quantitative cytopathology with reproducible model appraisal in a coherent teaching sequence. Nuclear feature distributions and importance patterns connect cellular morphology with diagnostic reasoning; classifier comparisons, calibration, and confusion matrices illustrate distinct dimensions of model performance; and alternative operating thresholds show how predictive probabilities translate into diagnostic decisions. Nested validation and reduced-feature analysis furnish practical examples for teaching data partitioning, feature redundancy, interpretability, and critical evaluation of medical AI. This shared analytical pathway provides case-based material for cytopathology, oncology, and interdisciplinary medical data science education.

## 1 Introduction

Breast cancer remains a major global public health challenge. According to the World Cancer Report, the number of people with cancer in all countries continues to rise due to an aging population and rising incidences (1, 2). Earlier discovery of malignant breast disease can reduce the time from abnormality to diagnosis, staging and treatment. Diagnostic pathways therefore need good sensitivity and specificity to avoid late diagnosis due to false negatives and unnecessary biopsies, anxiety and cost from false positives.

Cytopathology and image-derived quantitative morphology have been applied for a long time to assess breast masses. Fine-needle aspiration (FNA) obtains cells for examination by trained cytopathologists, and digitized cell nuclei can be used to acquire numerical descriptions such as radius, perimeter, area, smoothness, compactness, concavity, concave points, symmetry and fractal dimension. These descriptors are morphometric and textural abnormalities that are in line with malignant transformation, such as enlarged and irregular nuclei, heterogeneous chromatin, greater variation in nuclear shape, and disordered cellular structure. A well-known benchmark dataset for diagnostic models in breast cancer education and research is the public Wisconsin Diagnostic Breast Cancer (WDBC) dataset, which is based on digitized FNA samples (3, 4). Although it is not a replacement for the existing multi-center clinical dataset, an open and reproducible foundation for model testing in a controlled environment has been provided. Quantitative nuclear features also provide a structured entry point for cytopathology education. Nuclear enlargement, contour irregularity, heterogeneous texture, and altered symmetry can be introduced as morphological patterns and then examined through their corresponding measurements. The malignant and benign reference labels, together with explicitly defined feature families, support case-based comparison of individual measurements, feature distributions, and correlated descriptors. This progression links visual concepts of nuclear atypia with quantitative evidence and establishes a common basis for discussing how morphological abnormalities contribute to diagnostic classification.

Machine learning has been applied to cancer diagnosis, prognosis, treatment response prediction, radiomics, pathology image analysis and omics-based patient stratification (5–7). Technical feasibility is not sufficient; open reporting, uncertainty quantification, calibration assessment and clinically meaningful interpretation are also required. Performance in a single split may not be generalizable to different sample compositions, workflows or populations. Therefore, diagnostic-model research has gradually added repeated or stratified validation, probability calibration, clinical utility analysis and interpretable modeling. The updated Transparent Reporting of a multivariable prediction model for Individual Prognosis Or Diagnosis (TRIPOD) + Artificial Intelligence (AI) guidance, abbreviated TRIPOD+AI, recommends the following detailed reporting items: data sources, participants, candidate predictors, preprocessing, model development and evaluation, missing-data handling, and intended clinical context (8).

Recently, studies on breast cancer AI have used various machine-learning, ensemble and image-based approaches for different diagnostic data types (9–12). WDBC remains a public benchmark dataset, but recent comparative studies have demonstrated that the performance estimates are highly sensitive to feature selection, model combination and validation design (13). Explainable-AI research in breast cancer has further indicated that high discrimination should be accompanied by clinically meaningful interpretations of model predictions (14). Generally speaking, methodological studies of machine-learning clinical prediction models have repeatedly pointed out deficiencies in the reporting of internal validation and calibration (15). TRIPOD+AI guides the research, and the resulting evaluation system incorporates discrimination, calibration, uncertainty, interpretability and reproducibility to provide more comprehensive information rather than a single performance index. Teaching the evaluation of medical AI requires a similarly connected approach to model performance. ROC-AUC and precision-recall analysis characterize discrimination, whereas calibration examines the correspondence between predicted probabilities and observed outcomes. Confusion matrices and threshold-specific sensitivity and specificity then show how an operating rule influences diagnostic classifications. Examining these measures within the same dataset links statistical assessment to clinical reasoning and allows alternative diagnostic priorities to be compared through consistent predictions. The transparent validation procedure further provides a practical context for instruction in data partitioning, model selection, and reproducible reporting.

Recently, artificial intelligence in medical imaging has been developing at a high speed, and at present, many efforts have focused on directly learning from image pixels to locate and segment spatial lesions, while also considering computational efficiency and robustness. DeformNeXt-Swin integrates deformable convolutional processing with hierarchical Transformer-based contextual modeling in breast imaging to classify lesions in ultrasound and mammography and demonstrates how modern image-native models learn both local lesion morphology and broad tissue context directly from images (16). Correspondingly, many areas of medical imaging have also been extended to prediction problems. Recently, a large-scale comparison of YOLO-based models for localizing ischemic lesions in diffusion-weighted images of patients, along with computational-efficiency metrics and bootstrap uncertainty (17), and real-time YOLO-based colorectal polyp detection was also conducted across independent colonoscopy datasets, reporting parameters, computational operations and inference time (18). DiSCNet is a light-weight directional-convolutional network that achieves brain tumor classification by balancing diagnostic accuracy, computational efficiency, robustness to imaging heterogeneity, and visual explanation (19). GBA-Net extended image analysis to pixel-level ischemic-stroke segmentation of CT and DWI by combining gated contextual modeling with attention-refined feature fusion (20).

The above image-native studies are at the beginning of the diagnostic pipeline, and WDBC only contains numerical summaries of the digitized FNA nuclei. At present, the focus of this analysis is on the downstream diagnostic modeling of these pre-extracted cytopathology features under controlled internal validation, and it considers transparent coefficients, probability calibration, threshold selection, uncertainty, decision analysis and leakage-controlled feature evaluation. This feature-based perspective complements image-native research by focusing on the diagnostic interpretation of quantitative nuclear measurements within a transparent validation framework.

The combination of feature interpretation and diagnostic-model evaluation forms an instructional pathway spanning cytopathology and computational medicine. A case-based sequence can progress from characterizing nuclear morphology to comparing classifiers, interpreting held-out predictions, reviewing calibration, and examining threshold-dependent decisions. Feature reduction adds a complementary exercise in identifying informative yet correlated measurements and interpreting the relationship between model complexity and discrimination. By connecting these stages through one open dataset and an explicitly defined analytical workflow, the framework supports integrated teaching of pathological interpretation, diagnostic decision-making, and critical appraisal of medical AI.

Accordingly, this paper tests the hypothesis that a transparent linear diagnostic model constructed from pre-extracted cytopathology features can maintain high discrimination, stable coefficient directions, and performance-preserving dimensionality reduction when all data-dependent steps are restricted to training data. The analysis is framed as a journal research paper evaluating contemporaneous malignant-versus-benign diagnosis from cytopathology-derived measurements, rather than prognosis or prediction of a future clinical event. The retrospective, public, de-identified single-source dataset supports a controlled and reproducible internal benchmark while defining the scope of inference as internal diagnostic-model performance.

Clinically significant is not equivalent to high AUC in a benchmark set. Therefore, the above analysis compares several model families using cross-validation, evaluates their probability calibration and threshold suitability, assesses performance relative to the treat-all and treat-none reference strategies, determines whether a compact set of interpretable morphological features retains most diagnostic information, and investigates whether feature-importance patterns are cytopathologically plausible rather than the result of opaque artifacts.

Many oncology machine-learning studies have demonstrated overly optimistic performance indicators and lack in-depth reports on clinical application. A single accuracy value cannot demonstrate threshold-dependent predictions, probability calibration or improvement over the simple default strategy. The WDBC dataset offers a controlled environment for evaluating near-ceiling discrimination under an all-in, leakage-controlled system and can be used to investigate whether a transparent linear structure and a reduced feature panel maintain the original performance.

The principal contributions are as follows:

- A controlled public-data benchmark integrates data auditing, fold-contained preprocessing, model development, cross-validated evaluation, calibration assessment, exploratory decision analysis, and feature-level interpretation.
- Seven representative supervised-learning algorithms are compared within a common stratified cross-validation framework to support fair and reproducible evaluation of malignant-versus-benign breast mass classification.
- Full-feature and progressively reduced-feature strategies characterize the accumulation of diagnostic information and test whether a moderately smaller cytopathology feature panel preserves internal performance.
- Leakage-controlled validation performs feature ranking and operating-threshold optimization independently within outer-fold training data, while explicit mathematical definitions support reproducible calculation of the primary performance measures.
- Use of public, de-identified data supports an oncology-focused diagnostic-model analysis with transparent reporting of data availability, ethics, limitations, and reproducibility.

The rest of this paper is organized as follows. Section 2 introduces the research design, the WDBC dataset, cohort definition, candidate algorithms, cross-validation strategy, statistical indicators, leakage-controlled reduced-feature validation and ethics and reproducibility. Section 3 presents the experimental results, as well as cohort and feature characteristics, cross-validated model performance, calibration, threshold-specific behavior, decision-curve analysis, feature importance, reduced-feature robustness and fold-level stability. Section 4 introduces the diagnostic value, interpretability, methodological strengths and deficiencies, and demands for external validation of the study. Finally, Section 5 presents the main results and suggests further multicenter validation studies before clinical application.

## 2 Materials and methods

### 2.1 Study design

This paper reports a retrospective, non-interventional diagnostic model-development and internal-validation analysis using public, de-identified WDBC data. The target is the contemporaneous diagnosis/classification of the sampled breast mass as malignant or benign from cytopathology-derived nuclear features; the analysis does not predict prognosis, recurrence, future disease risk, treatment response, or another longitudinal outcome. **Figure 1** demonstrates the study workflow, including data auditing and preprocessing, nested model development, out-of-fold performance assessment, calibration and threshold analysis, decision-curve analysis, feature interpretation, and reduced-feature sensitivity analysis. Malignancy was prespecified as the positive class. Preprocessing parameters and model hyperparameters were estimated within each outer-fold training partition. Continuous-metric evaluation used held-out outer-fold probabilities. Operating thresholds were estimated separately inside each outer-fold training partition from inner-fold out-of-fold probabilities and were locked before application to the corresponding held-out outer fold. The ordered stages of nuclear-feature assessment, nested validation, and threshold-specific prediction also form a reproducible sequence for practical instruction in diagnostic-model evaluation.

**Figure 1:**
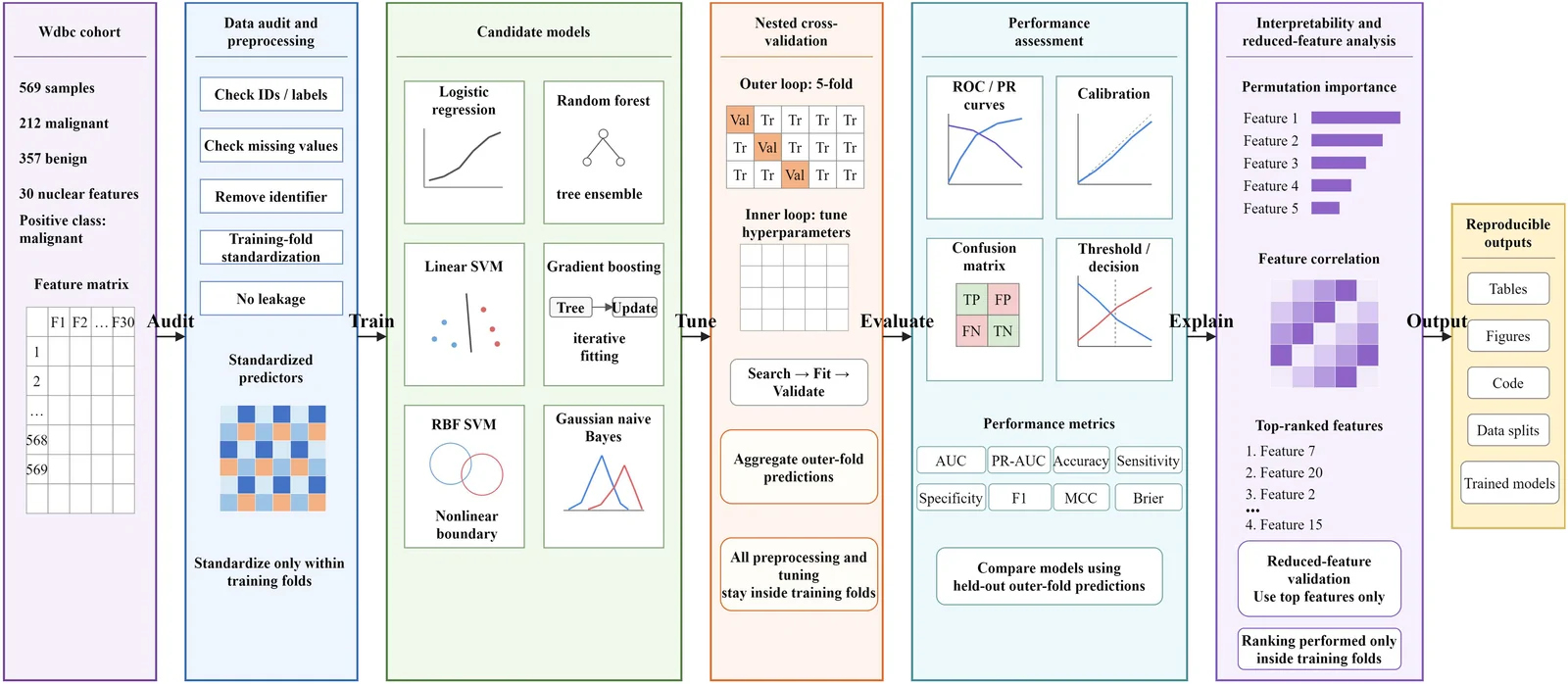
Reproducible study workflow. The diagram summarizes the WDBC cohort (n=569; 212 malignant and 357 benign), fold-contained preprocessing, nested model development and validation, discrimination and calibration assessment, decision-curve analysis, feature interpretation, and reporting outputs.

### 2.2 Data source and cohort definition

The Wisconsin Diagnostic Breast Cancer (WDBC) dataset was downloaded from the UCI Machine Learning Repository and contains 569 digitized fine-needle aspiration (FNA) breast-mass samples with 30 continuous nuclear features (4). The original Wisconsin work demonstrated diagnostic separation using FNA-derived cellular features (21). The 30 predictors arise from ten nuclear characteristics, each represented by mean, standard-error, and worst-value summaries; the worst value is the mean of the three largest measurements. Malignancy was the positive class, with 212 malignant and 357 benign samples (37.3% prevalence), and the standard release contained no missing values. Scale-sensitive algorithms used standardization parameters estimated only from the corresponding training fold, whereas tree-based models used the original feature scales; identical cross-validation partitions were used for all models. Data auditing checked duplicate identifiers, missing labels, missing feature values, and invalid class codes in line with the participant/data-source, predictor, outcome, and analysis domains of the Prediction model Risk Of Bias Assessment Tool (PROBAST) (22). The ‘id’ column was excluded from the predictors, and fold-specific standardization and model fitting were implemented in scikit-learn pipelines to avoid information leakage (23).

As defined in the UCI WDBC release (4), the ten underlying nuclear characteristics are: radius (mean distance from the nuclear center to perimeter points), texture (standard deviation of gray-scale values), perimeter, area, smoothness (local variation in radius lengths), compactness (perimeter squared divided by area minus 1.0), concavity (severity of concave contour portions), concave points (number of concave contour portions), symmetry, and fractal dimension (coastline approximation minus 1). The complete 30-feature set is: mean radius, mean texture, mean perimeter, mean area, mean smoothness, mean compactness, mean concavity, mean concave points, mean symmetry, mean fractal dimension; radius error, texture error, perimeter error, area error, smoothness error, compactness error, concavity error, concave points error, symmetry error, fractal dimension error; and worst radius, worst texture, worst perimeter, worst area, worst smoothness, worst compactness, worst concavity, worst concave points, worst symmetry, and worst fractal dimension. In the analysis, the ‘error’ fields correspond to the standard-error summaries supplied by the dataset. For cytopathology education, these quantitative descriptors can be organized into three interrelated morphological domains: nuclear size (radius, perimeter, and area), contour irregularity (concavity and concave points), and texture or shape (texture, symmetry, smoothness, compactness, and fractal dimension). The corresponding mean, standard-error, and worst-value summaries provide a structured basis for comparing typical nuclear morphology, within-sample variability, and pronounced nuclear atypia in malignant and benign samples.

**Figure 2** demonstrates the class composition of the WDBC cohort: 357 benign samples (62.7%) and 212 malignant samples (37.3%). Because this moderate imbalance can make accuracy alone incomplete, evaluation also included sensitivity, specificity, precision, F1-score, Matthews correlation coefficient (MCC), ROC-AUC, PR-AUC, and Brier score.

**Figure 2:**
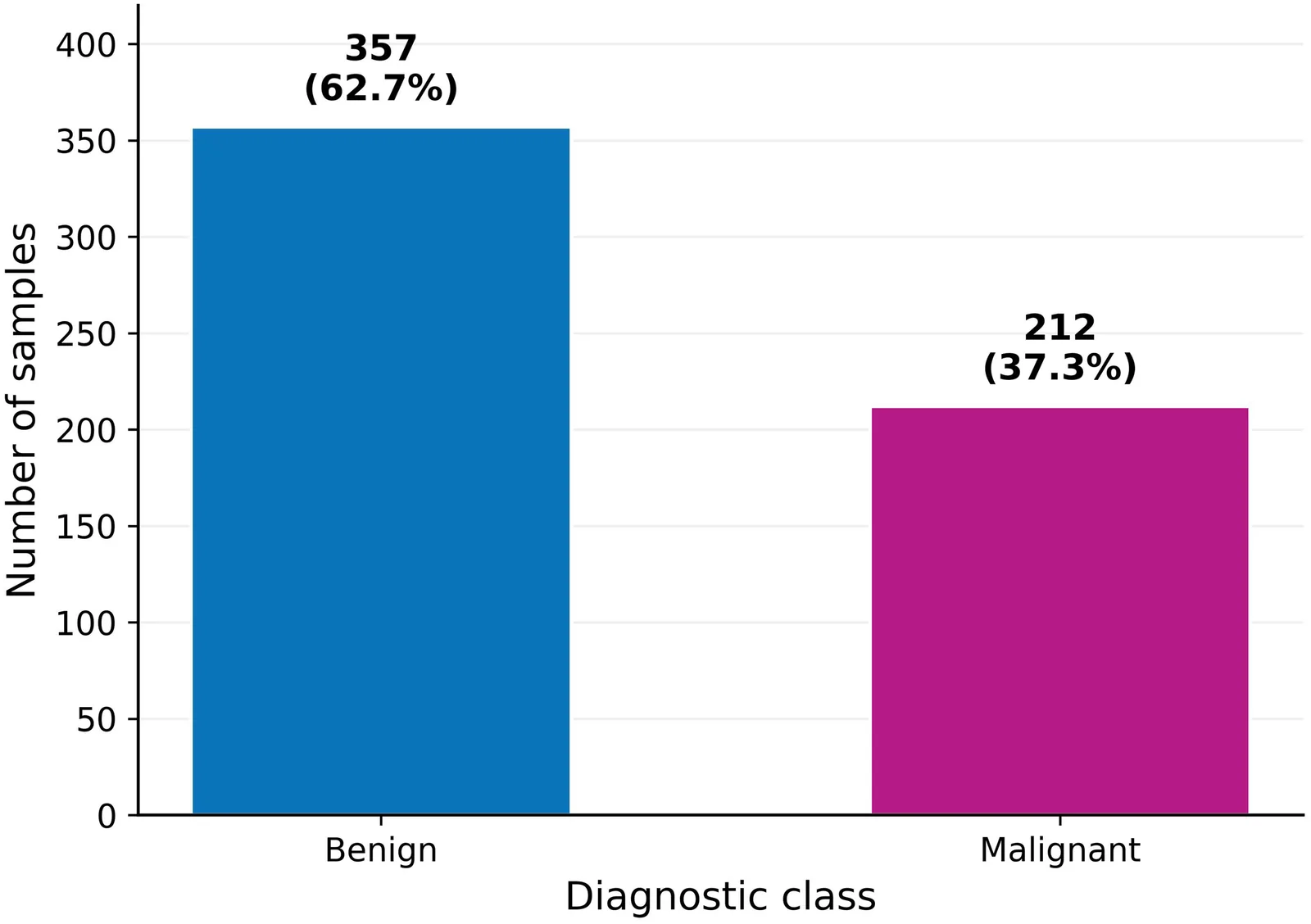
Diagnostic class distribution in the open WDBC cohort (n=569). Bars display 357 benign samples (62.7%) and 212 malignant samples (37.3%); malignancy is the positive class in all analyses.

**Figure 3** demonstrates pairwise Pearson correlations among 12 informative cytopathology features. The strong correlations among several size- and contour-related descriptors motivate interpretation at the feature-family level and support the complementary full-feature and reduced-feature analyses used later in this paper.

**Figure 3:**
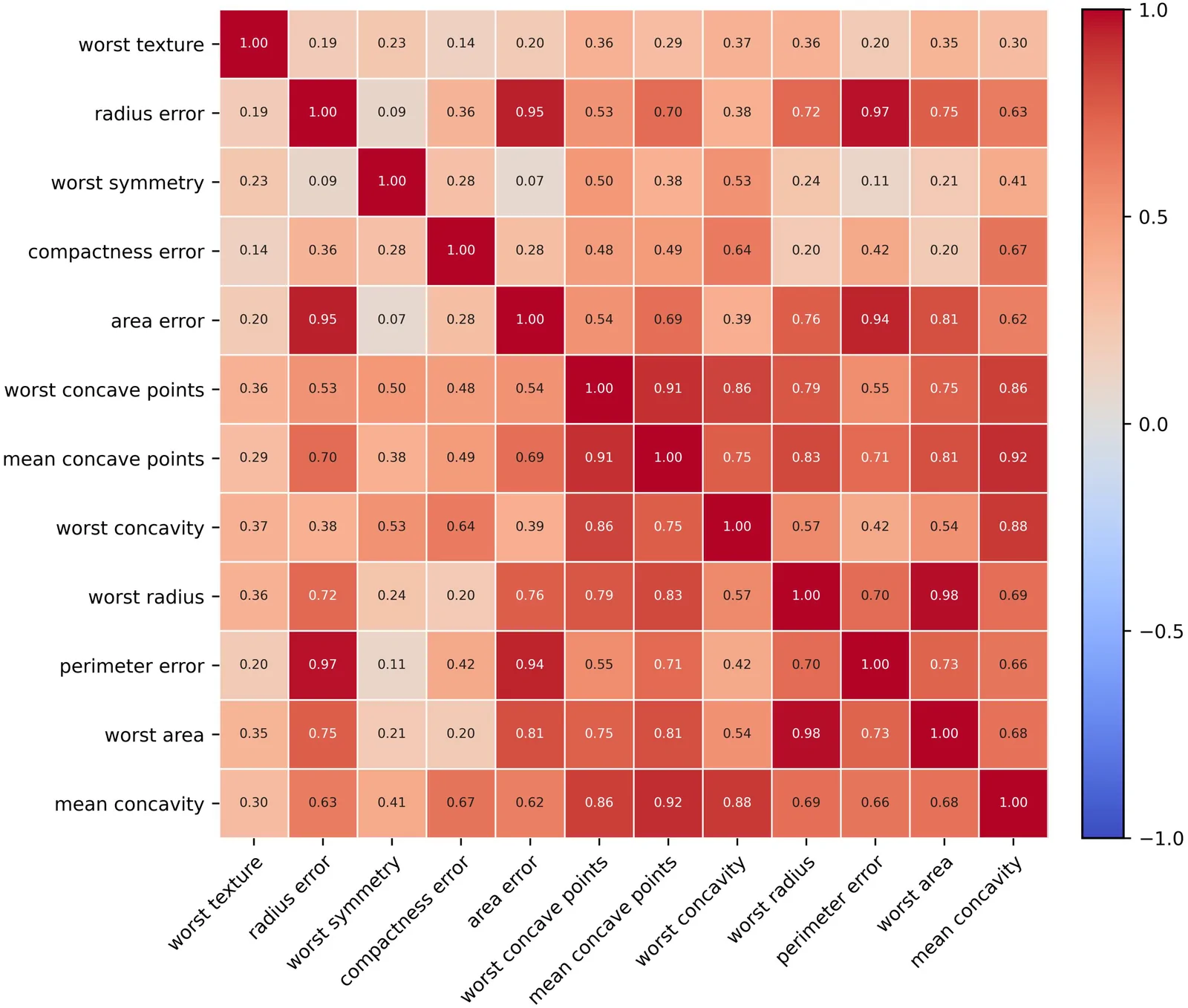
Pearson correlation matrix for 12 informative cytopathology features in the WDBC cohort (n=569). Cell values and color intensity indicate pairwise correlations, with red denoting positive and blue denoting negative association.

**Table 1** summarizes cohort composition, preprocessing, missing-data status, and validation design, which provide context for interpreting discrimination, calibration, and threshold-dependent performance.

**Table 1:** Dataset characteristics and preprocessing strategy.

Item	Value or procedure	Analytical implication
Total samples	569	Adequate for methodological benchmarking but limited for definitive clinical deployment.
Malignant samples	212 (37.3%)	Positive diagnostic class for sensitivity and precision calculations.
Benign samples	357 (62.7%)	Reference negative class for specificity calculations.
Predictors	30 continuous cytomorphometric features: mean, standard-error, and worst summaries for 10 nuclear characteristics (radius, texture, perimeter, area, smoothness, compactness, concavity, concave points, symmetry, and fractal dimension).	The complete 30-feature names are specified explicitly in Section 2.2; all are continuous predictors derived from the same digitized FNA workflow.
Missing values	None in the standard release	No imputation was required.
Scaling	Training-fold standardization for scale-sensitive models	Prevents data leakage from test folds.
Validation	Nested stratified cross-validation: five outer folds and four inner folds	Separates held-out evaluation from hyperparameter and threshold selection.

### 2.3 Candidate models and training protocol

Seven candidate algorithms represented complementary model classes: regularized logistic regression, calibrated linear SVM, RBF SVM, random forest, gradient boosting, k-nearest neighbors, and Gaussian Naive Bayes. Logistic regression and linear SVM provided interpretable linear baselines; RBF SVM assessed nonlinear separation; random forest and gradient boosting represented ensemble methods; k-nearest neighbors evaluated local similarity; and Gaussian Naive Bayes provided a probabilistic benchmark (24–27).

Given the cohort size (n=569) and 30 structured predictors, the comparison emphasized model classes with controlled complexity and established applicability to tabular data. When predictive performance is comparable, simpler decision structures also facilitate direct interpretation in diagnostic modeling.

All models were evaluated using nested cross-validation. The outer loop used stratified five-fold splits generated with random seed 42 and supplied the held-out predictions used for performance estimation. Within each outer training partition, a separate stratified four-fold inner loop performed grid-search hyperparameter optimization using ROC-AUC as the selection score. Standardization, model fitting, hyperparameter selection, and SVM probability calibration were confined to the corresponding outer training partition. After hyperparameter selection, inner-fold out-of-fold probabilities from that outer training partition were used to select its Youden-balanced and sensitivity-prioritized thresholds. The selected pipeline and the two fold-specific thresholds were then applied to the corresponding held-out outer validation fold. Concatenating the five held-out probability vectors produced one out-of-fold probability for each sample for discrimination, calibration, precision-recall, decision-curve, and paired statistical analyses; threshold-specific metrics were calculated from the concatenated fold-specific held-out decisions.

Grid search used the following prespecified candidate sets: logistic-regression and linear-SVM regularization C in {0.001, 0.003, 0.01, 0.03, 0.1, 0.3}; RBF-SVM C in {0.1, 0.3, 1, 3, 10} and gamma in {scale, 0.001, 0.01, 0.1}; random-forest trees in {150, 300}, maximum depth in {unrestricted, 8}, and minimum leaf size in {1, 2}; gradient-boosting trees in {100, 200}, learning rate in {0.03, 0.05, 0.1}, and maximum depth in {1, 2}; KNN neighbors in {5, 7, 9, 11}, weighting in {uniform, distance}, and Minkowski power in {1, 2}; and Gaussian-naive-Bayes variance smoothing in {1e-11, 1e-10, 1e-9, 1e-8, 1e-7}. Balanced class weights were fixed for logistic regression and both SVMs, balanced subsampling and square-root feature sampling were fixed for random forest, and sigmoid probability calibration for the SVMs was fitted within outer training data only.

ROC-AUC was used as the primary threshold-independent discrimination measure. Threshold-specific performance was evaluated at the default threshold of 0.50 and with strictly nested fold-specific Youden and sensitivity-prioritized thresholds using accuracy, sensitivity, specificity, precision, F1-score, and MCC. PR-AUC was reported to characterize positive-class retrieval at the observed malignant-case prevalence, and the Brier score quantified probabilistic prediction error.

**Table 2** summarizes the candidate algorithms, prespecified configurations, and rationale for inclusion in the comparative analysis. Representative or modal settings across outer folds are reported, reflecting fold-specific hyperparameter selection. In outer-fold order, selected C values were 0.1, 0.1, 0.3, 0.1, and 0.3 for logistic regression, and 0.01, 0.01, 0.1, 0.1, and 0.03 for the linear SVM. Logistic-regression C = 0.1 occurred in three folds and C = 0.3 in two; linear-SVM C = 0.01 and C = 0.1 each occurred in two folds and C = 0.03 in one.

**Table 2:** Candidate models and modeling assumptions.

Model	Representative outer-fold setting and fixed assumptions	Reason for inclusion
Logistic regression	C=0.1 (modal); L2 regularization, balanced class weights, standardized features	Interpretable linear diagnostic baseline.
Linear SVM	C=0.01 and 0.1 (tied modal); sigmoid-calibrated linear kernel, balanced class weights	Tests linear separability with margin maximization.
RBF SVM	C=3 and gamma=0.01 (modal); balanced class weights, standardized features	Captures nonlinear feature interactions.
Random forest	150 trees, unrestricted depth, minimum leaf size 1 (modal); balanced subsampling	Robust ensemble baseline with nonlinear splits.
Gradient boosting	200 trees, learning rate 0.1, depth 2 (modal)	Sequential additive model for nonlinear decision boundaries.
KNN	11 distance-weighted neighbors, Minkowski power 2 (modal); standardized features	Assesses local cytomorphometric similarity.
Gaussian naive Bayes	Variance smoothing 1e-11 (selected in all outer folds)	Simple probabilistic benchmark.

For the RBF SVM, selected (C, gamma) pairs were (3, 0.01), (1, 0.01), (3, 0.01), (3, 0.01), and (3, 0.01). Random-forest (trees, maximum depth, minimum leaf size) selections were (150, 8, 1), (300, unrestricted, 1), (300, unrestricted, 2), (150, unrestricted, 1), and (150, unrestricted, 1). Gradient-boosting (trees, learning rate, depth) selections were (200, 0.1, 2), (200, 0.05, 2), (200, 0.1, 1), (200, 0.1, 2), and (200, 0.1, 1). KNN (neighbors, weighting, Minkowski power) selections were (11, uniform, 2), (11, distance, 2), (9, distance, 2), (11, distance, 2), and (11, distance, 2). Gaussian-naive-Bayes variance smoothing was 1e-11 in all five folds. The modal RBF pair occurred in four folds; the random-forest modal tree count, depth, and leaf size occurred in three, four, and four folds; gradient boosting selected 200 trees in all folds, learning rate 0.1 in four, and depth 2 in three; KNN selected 11 neighbors in four, distance weighting in four, and power 2 in all folds.

### 2.4 Statistical analysis and performance metrics

For each sample, the model returned a probability of malignancy, with the reference label coded as y = 1 for malignant and y = 0 for benign. ROC-AUC and average precision assessed discrimination (28, 29), and threshold-based predictions were evaluated using sensitivity, specificity, precision, accuracy, F1-score, and MCC. Formal pairwise comparison focused on the linear SVM, RBF SVM, and logistic regression. Correlated ROC-AUCs were compared using two-sided DeLong tests, and paired classifications at 0.50 were compared using two-sided exact McNemar tests. A p-value below 0.05 was considered statistically significant. Calibration was quantified from outer-fold out-of-fold probabilities using the Brier score, calibration intercept, and calibration slope; ideal values are 0 for the intercept and 1 for the slope. The intercept and slope were estimated by logistic recalibration of the observed outcome on the logit of the predicted probability, with uncertainty estimated by stratified bootstrap resampling conditional on the fixed out-of-fold predictions (30). Decision-curve analysis was used exploratorily and modeled escalation to expert cytopathology review and/or confirmatory tissue examination as the clinical action. Net benefit was evaluated across threshold probabilities from 0.05 to 0.95 to demonstrate how estimated utility changes across a broad spectrum of relative harm preferences rather than at one arbitrary fixed value. This decision-curve threshold probability is conceptually distinct from the operating classification threshold used to convert predicted probabilities into malignant/benign labels. Interpretation was prespecified primarily for 0.05-0.30, where the implied relative weight of a missed malignancy versus unnecessary escalation changes from approximately 19 to 2.33; thresholds above 0.30 were displayed to document the full net-benefit trajectory and were not treated as a single recommended operating point (31). Permutation importance was defined as the reduction in ROC-AUC after randomly permuting a feature. Because correlated cytomorphometric variables can share predictive information, interpretation emphasized feature groups and contextualized individual rankings within correlated morphology domains (32). Class distributions of the leading variables were compared using standardized mean differences and reduced-feature sensitivity analysis evaluated logistic-regression models with the top 3, 5, 8, 10, 15, 20, and 30 mutual-information-ranked features. The evaluation metrics were organized into three complementary dimensions of diagnostic-model assessment: discrimination, represented by ROC-AUC and PR-AUC; probability reliability, characterized by calibration measures and the Brier score; and decision consequences, reflected by sensitivity, specificity, confusion matrices, and decision-curve net benefit. This integrated evaluation structure provides a reproducible basis for teaching the distinctions among model discrimination, probability estimation, and threshold-dependent diagnostic decision-making.

The computing protocol set a fixed random seed of 42 for cross-validation partitioning, model stochastic components, bootstrap resampling and permutation importance. Linear SVM probabilities were obtained after sigmoid calibration, and calibration was fitted only on the training data of the corresponding cross-validation fold.

Within each outer fold, the Youden-balanced threshold was selected over a 0.001-spaced grid from 0.001 to 0.999 by maximizing the Youden index defined below using only inner-fold out-of-fold probabilities from that outer training partition. After hyperparameter selection, the sensitivity-prioritized threshold was defined as the highest grid value achieving training sensitivity of at least 0.99, which also maximized specificity among feasible grid values. Both thresholds were fixed for the corresponding fold and applied to its held-out outer validation data. Aggregate threshold-specific metrics and confusion-matrix counts were calculated after concatenating the five sets of held-out decisions.

For the linear SVM, uncertainty was quantified using 1,000 stratified sample-level bootstrap resamples of the fixed outer-fold out-of-fold predictions and the corresponding fold-specific decisions. Malignant and benign samples were resampled separately with replacement, and two-sided 95% percentile confidence intervals were defined by the 2.5th and 97.5th percentiles. The conditional estimand characterizes sampling variation in the fitted out-of-fold predictions and selected fold-specific thresholds. ROC-AUC, PR-AUC, Brier score, calibration intercept, and calibration slope were recalculated from continuous probabilities in each replicate, whereas threshold-dependent metrics used the corresponding held-out decisions.

The equations below use the sample index i, feature index j, and threshold t consistently. Unless otherwise stated, reported performance statistics are calculated from held-out predictions. Fold-contained standardization is defined by Equation 1:

(1) $$z_{ij}=\frac{x_{ij}-\mu_{j}^{train}}{\sigma_{j}^{train}}$$

where *x_ij_* denotes the original value of feature *j* in sample *i*, *z_ij_* denotes the standardized value used by scale-sensitive algorithms, and $\mu_{j}^{train}$ and $\sigma_{j}^{train}$ denote the mean and standard deviation of feature *j* estimated from the training fold only. All training-specific location and scale terms are estimated only from the corresponding training fold.

The predicted probability that sample i belongs to the malignant class is given by Equation 2:

(2) $$p_{i}=P\left(y_{i}=1|X_{i}\right)$$

where *p_i_* denotes the predicted probability of malignancy for sample *i*, *y_i_* denotes the reference label, and *X_i_* denotes the full predictor vector for sample *i*. This probability represents contemporaneous diagnostic classification, not future-event risk.

After a threshold has been fixed from training data, the continuous probability is converted to a binary diagnostic label by Equation 3:

(3) $${\hat{y}}_{i}=\{\begin{array}{ll} 1, & p_{i}\geq t\\ 0, & p_{i}<t \end{array}$$

where ${\hat{y}}_{i}$ denotes the predicted diagnostic class for sample *i*, and *t* denotes the probability threshold used to classify a sample as malignant. The threshold is locked before evaluation on the corresponding held-out outer fold.

Sensitivity and specificity at threshold t are calculated using Equation 4:

(4) $$\text{Se}\left(t\right)\;=\;\frac{\text{TP}\left(t\right)}{\text{TP}\left(t\right)+\text{FN}\left(t\right)},\text{ Sp}\left(t\right)=\frac{\text{TN}\left(t\right)}{\text{TN}\left(t\right)+\text{FP}\left(t\right)}$$

where Se(*t*) and Sp(*t*) denote sensitivity and specificity at threshold *t*, and TP(*t*), FN(*t*), TN(*t*), and FP(*t*) denote the numbers of true positives, false negatives, true negatives, and false positives, respectively. Sensitivity quantifies malignant-case detection, whereas specificity quantifies correct rejection of benign cases.

Precision and accuracy at threshold t are calculated using Equation 5:

(5) $$\text{PPV}\left(t\right)\;=\;\frac{\text{TP}\left(t\right)}{\text{TP}\left(t\right)+\text{FP}\left(t\right)},\text{ Acc}\left(t\right)=\frac{\text{TP}\left(t\right)+\text{TN}\left(t\right)}{\text{TP}\left(t\right)+\text{TN}\left(t\right)+\text{FP}\left(t\right)+\text{FN}\left(t\right)}$$

where PPV(*t*) denotes precision or positive predictive value at threshold *t*, and Acc(*t*) denotes the overall proportion of correctly classified samples. Both metrics are computed from the same held-out thresholded decisions.

The F1-score, combining precision and sensitivity through their harmonic mean, is given by Equation 6:

(6) $$F1\left(t\right)=\frac{2\times \text{PPV}\left(t\right)\times \text{Se}\left(t\right)}{\text{PPV}\left(t\right)+\text{Se}\left(t\right)}$$

where *F*1(*t*) denotes the harmonic mean of precision and sensitivity at threshold *t*. Higher F1 values indicate a better balance between positive predictive value and malignant-case sensitivity.

The Matthews correlation coefficient (MCC), which incorporates all four confusion-matrix cells, is given by Equation 7:

(7) $$\text{MCC}\left(t\right)=\frac{\text{TP}\left(t\right)\times \text{TN}\left(t\right)-\text{FP}\left(t\right)\times \text{FN}\left(t\right)}{\sqrt{\left(\text{TP}\left(t\right)+\text{FP}\left(t\right)\right)\left(\text{TP}\left(t\right)+\text{FN}\left(t\right)\right)\left(\text{TN}\left(t\right)+\text{FP}\left(t\right)\right)}\left(\text{TN}\left(t\right)+\text{FN}\left(t\right)\right)}$$

where MCC(*t*) denotes a balanced correlation-based summary of binary classification performance at threshold *t*. MCC ranges from -1 to 1 and remains informative when class frequencies are unequal.

The Youden index used for balanced operating-threshold selection is by Equation 8:

(8) $$\text{J}\left(t\right)=\text{Se}\left(t\right)+\text{Sp}\left(t\right)-1,\;t_{\text{opt}}=\underset{t}{\arg\;\max}J\left(t\right)$$

where J(*t*) denotes the Youden index at threshold *t*, and *t*_opt_ denotes the threshold that maximizes J(*t*) within the evaluated threshold grid. In this paper, the maximization is performed only with training-derived inner-fold probabilities.

The Brier score used to quantify probabilistic prediction error is Equation 9:

(9) $$\text{Brier}=\frac{1}{n}\sum_{i=1}^{n}{\left(p_{i}-y_{i}\right)}^{2}$$

where *n* denotes the total number of samples, *p_i_* denotes the predicted probability of malignancy, and *y_i_* denotes the reference diagnostic label. Lower Brier scores indicate smaller squared probabilistic error.

Exploratory decision-curve net benefit at threshold probability t is given by Equation 10:

(10) $$\text{NB}\left(t\right)=\frac{\text{TP}\left(t\right)}{n}-\frac{\text{FP}\left(t\right)}{n}\times \frac{t}{1-t}$$

where NB(*t*) denotes the net benefit at threshold probability *t*, and the term $t/\left(1-t\right)$ represents the relative weight assigned to false-positive classifications. In Equation 10, t is the decision-curve threshold probability and must not be confused with a single deployed classification threshold; varying t is necessary to examine net benefit under different false-positive/false-negative trade-offs.

Permutation importance for feature j is defined by Equation 11 as the loss of discrimination after permutation:

(11) $${\text{PI}}_{j}={\text{AUC}}_{\text{orig}}-{\text{AUC}}_{\text{perm},j}$$

where PI*_j_* denotes the permutation importance of feature *j*, ${\text{AUC}}_{\text{orig}}$ denotes the AUC before permutation, and ${\text{AUC}}_{\text{perm},j}$ denotes the AUC after randomly permuting feature *j*. A positive permutation-importance value indicates that disrupting feature j reduces ROC-AUC.

The standardized mean difference for feature j is used to quantify between-class separation, as shown in Equation 12:

(12) $${\text{SMD}}_{j}=\frac{{\bar{x}}_{M,j}-{\bar{x}}_{B,j}}{\sqrt{\left(s_{M,j}^{2}+s_{B,j}^{2}\right)/2}}$$

where SMD*_j_* denotes the standardized mean difference for feature *j*, ${\bar{x}}_{M,j}$ and ${\bar{x}}_{B,j}$ denote malignant-class and benign-class means, and $s_{M,j}$ and $s_{B,j}$ denote malignant-class and benign-class standard deviations. The pooled within-class variability in the denominator makes the measure scale-independent.

### 2.5 Leakage-controlled reduced-feature validation

Leakage-controlled reduced-feature validation assessed whether reduced-feature performance remained stable when predictor ranking was restricted to the training data within each outer fold (33–35). Feature ranking was nested within the outer stratified five-fold cross-validation loop. For each outer fold, mutual-information scores were calculated exclusively from the training samples, which determined the top 3, 5, 8, 10, 15, 20, and 30 feature panels. A balanced logistic-regression model was fitted on the selected training-fold features and applied to the corresponding held-out validation fold. Repeating this procedure across all outer folds yielded one out-of-fold probability per sample for the reported performance measures. Selection frequency within fold-specific top-10 panels assessed interpretive stability. Paired stratified bootstrap resampling of the fixed out-of-fold predictions with 2,000 replicates quantified ROC-AUC differences for the 20-versus-30 and 10-versus-30 feature comparisons. Repeated training-fold feature ranking and held-out evaluation establish a traceable analytical sequence for examining the relationship between predictor selection, model complexity, and diagnostic discrimination. This process also provides a practical framework for teaching information-leakage prevention, dimensionality reduction, and the methodological principles of reliable internal validation.

### 2.6 Ethics and reproducibility considerations

The use of exclusively public, de-identified secondary data met the criteria for exemption from ethics committee review. Reporting was guided by TRIPOD+AI, and the Prediction model Risk Of Bias Assessment Tool (PROBAST) framework informed evaluation of potential bias across participants/data sources, predictors, outcomes, and analysis. Open-source Python libraries were used for modeling and visualization to support reproducibility.

Reporting covered data source and eligibility, outcomes and predictors, missing data, fold-contained preprocessing, model specification and tuning, internal validation, uncertainty, calibration, intended use, limitations, and data/code availability in accordance with TRIPOD+AI. Relevant CLAIM principles informed transparency for the use of pre-extracted image-derived features (36), while CONSORT-AI and SPIRIT-AI provide trial-oriented context for prospective evaluation (37, 38). Established clinical-AI guidance supported interpretation of representativeness, workflow context, calibration, and external validation (39–43). Stratified cross-validation and bootstrap estimation supported internal validation (44), correlated ROC comparisons followed the standard nonparametric AUC framework (45), and feature distributions, permutation importance, and leakage-controlled reduced-feature models supported cytopathological interpretation (46–48).

Analyses were performed in Python 3.12.13 using scikit-learn 1.8.0, NumPy 2.3.5, pandas 2.2.3, SciPy 1.17.0, matplotlib 3.10.8, and seaborn 0.13.2. Reproducibility was supported by fixed random seeds, version-pinned dependencies, analysis scripts, and documented procedures for generating the reported tables and figures.

## 3 Results

### 3.1 Cohort composition and feature structure

The cohort of 569 breast-mass samples comprised 212 malignant (37.3%) and 357 benign (62.7%) cases. **Figure 2** demonstrates the resulting moderate class imbalance and provides context for reporting sensitivity, specificity, PR-AUC, MCC, and accuracy together. **Figure 3** demonstrates that several size-related and boundary-irregularity variables are strongly correlated. Radius, perimeter, and area capture overlapping aspects of nuclear size, whereas concavity and concave-point measures capture related aspects of boundary irregularity. This redundancy supports interpretation at the feature-family level and motivates reduced-feature analysis.

### 3.2 Cross-validated model discrimination and classification performance

**Table 3** summarizes outer-fold out-of-fold performance for all seven candidate models and paired difference tests comparing the linear SVM with the RBF SVM and logistic regression. ROC-AUC and PR-AUC describe discrimination, while threshold-specific metrics and the Brier score characterize classification and probabilistic performance at the prespecified threshold of 0.50.

**Table 3:** Comparative nested cross-validated outer-fold performance of the seven candidate models at the default probability threshold of 0.50 and pairwise comparisons with the linear SVM (n = 569).

Model	AUC	PR-AUC	Accuracy	Sensitivity	Specificity	F1	MCC	Brier	DeLong p-value*	McNemar p-value^†^
Linear SVM	0.994	0.993	0.979	0.958	0.992	0.971	0.955	0.021	—	—
RBF SVM	0.995	0.994	0.981	0.972	0.986	0.974	0.959	0.020	0.43	1
Logistic regression	0.994	0.993	0.975	0.967	0.980	0.967	0.947	0.023	0.829	0.688
Random forest	0.989	0.987	0.961	0.943	0.972	0.948	0.917	0.033	—	—
Gradient boosting	0.991	0.987	0.954	0.920	0.975	0.938	0.902	0.031	—	—
KNN	0.990	0.988	0.967	0.925	0.992	0.954	0.929	0.030	—	—
Gaussian NB	0.987	0.980	0.933	0.863	0.975	0.906	0.857	0.062	—	—

*DeLong p-values test differences in correlated ROC-AUCs relative to the linear SVM.

^†^Exact McNemar p-values test differences in paired classifications relative to the linear SVM at 0.50. Non-significant p-values indicate limited evidence of a classification difference. Pairwise inference focused on the RBF SVM and logistic regression as the two closest-performing alternatives.

All seven models achieved high discrimination, with ROC-AUC values from 0.987 to 0.995 and PR-AUC values from 0.980 to 0.994. The RBF SVM was the numerical leader for ROC-AUC, PR-AUC, accuracy, sensitivity, F1-score, and MCC at 0.50, while the linear SVM and KNN achieved the highest specificity. DeLong comparisons yielded p=0.430 for the linear versus RBF SVM and p=0.829 for the linear SVM versus logistic regression; exact McNemar tests at 0.50 yielded p=1.000 and p=0.688, respectively. These results indicate limited evidence of pairwise performance differences in this sample. The RBF SVM remained the numerical discrimination leader, and the linear SVM served as the interpretable analysis model for threshold and coefficient evaluation because of its high specificity and directly inspectable linear decision structure.

**Figure 4** demonstrates the outer-fold out-of-fold ROC curves for all seven candidate models. The curves cluster near the upper-left corner, and the small separation among the leading models is consistent with the pairwise DeLong results, indicating strong shared discrimination from the cytomorphometric feature set.

**Figure 4:**
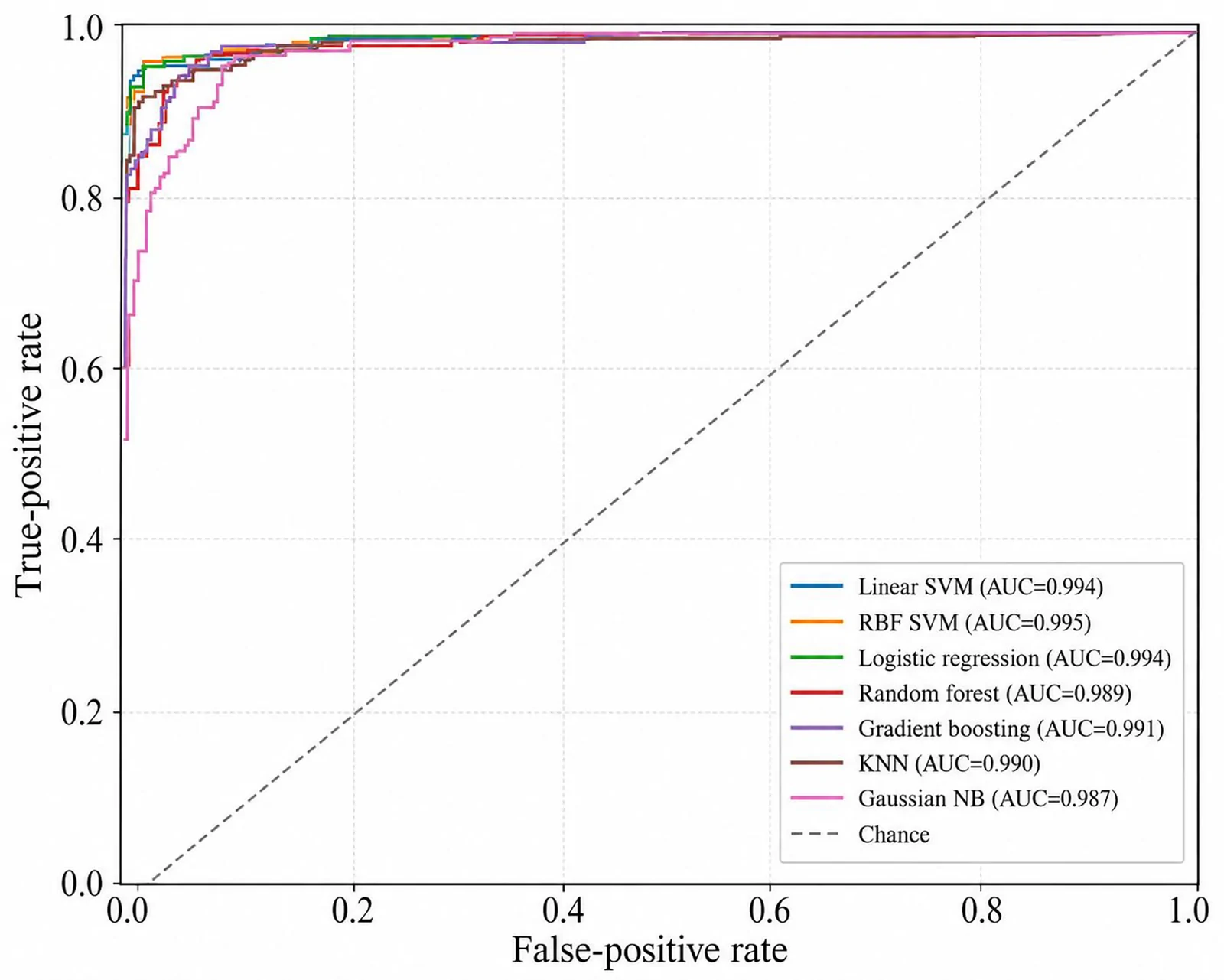
Outer-fold out-of-fold receiver operating characteristic curves for seven candidate models (n=569). Malignancy is the positive class, ROC-AUC values are reported in the legend, and the diagonal represents chance discrimination.

**Figure 5** separately demonstrates precision-recall performance for malignant-class prediction. At the observed malignant prevalence of 37.3%, the leading models maintain high precision across most of the recall range, complementing the ROC analysis with a positive-class retrieval perspective.

**Figure 5:**
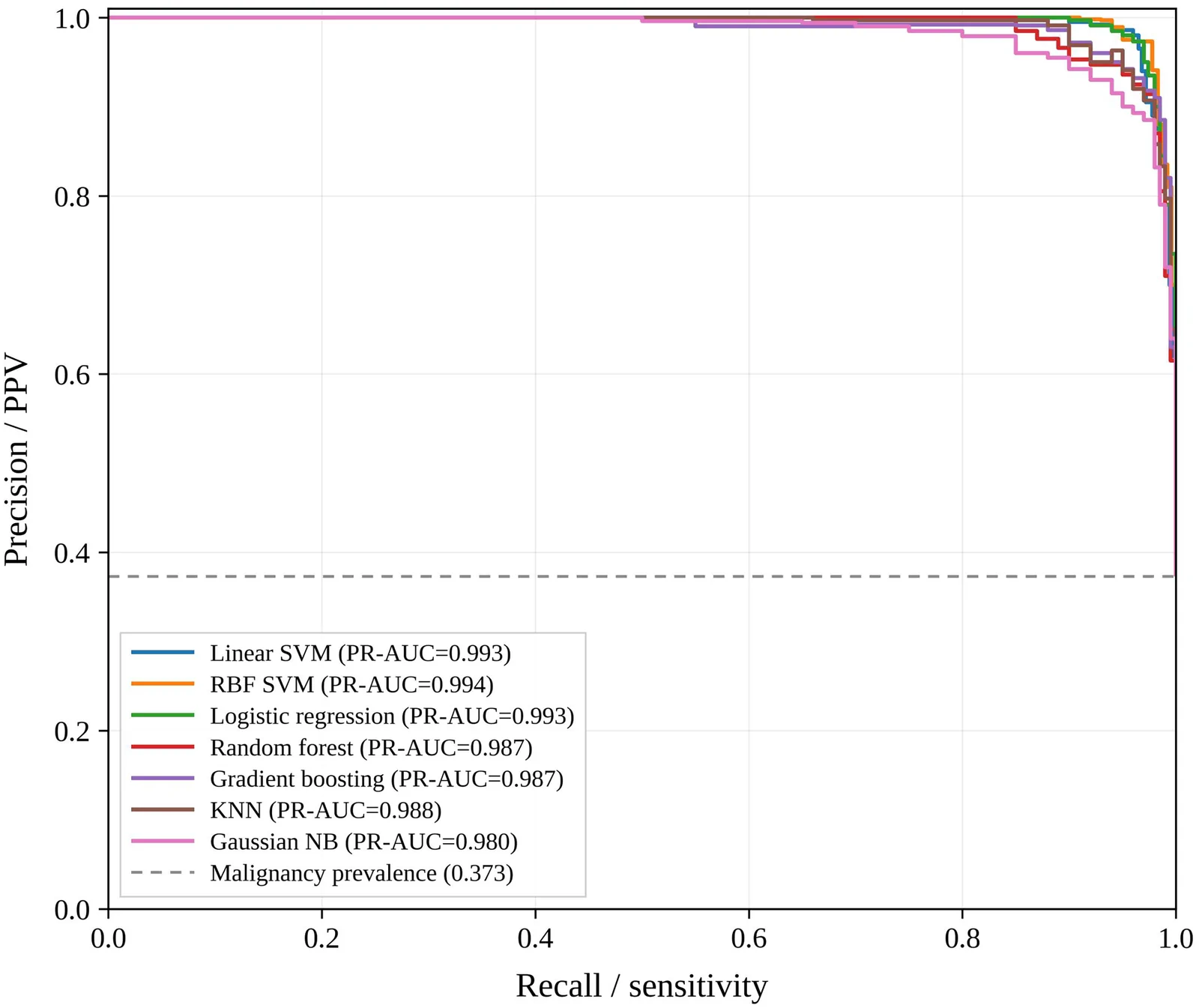
Outer-fold out-of-fold precision-recall curves for malignant-class prediction (n=569). PR-AUC values are reported in the legend, and the horizontal reference line denotes the malignant-class prevalence of 37.3%.

### 3.3 Calibration and threshold-specific diagnostic behavior

**Figure 6** demonstrates calibration curves derived from outer-fold out-of-fold probabilities. For the linear SVM, the Brier score was 0.021, calibration intercept was 0.280 (95% conditional bootstrap CI -0.164 to 0.867), and calibration slope was 1.284 (1.004 to 2.004). The intercept interval included 0 and the slope estimate exceeded 1 with substantial uncertainty, indicating favorable internal calibration with residual uncertainty in probability scaling. Local deviations were greatest in sparsely populated extreme-risk bins, supporting independent calibration assessment across external populations.

**Figure 6:**
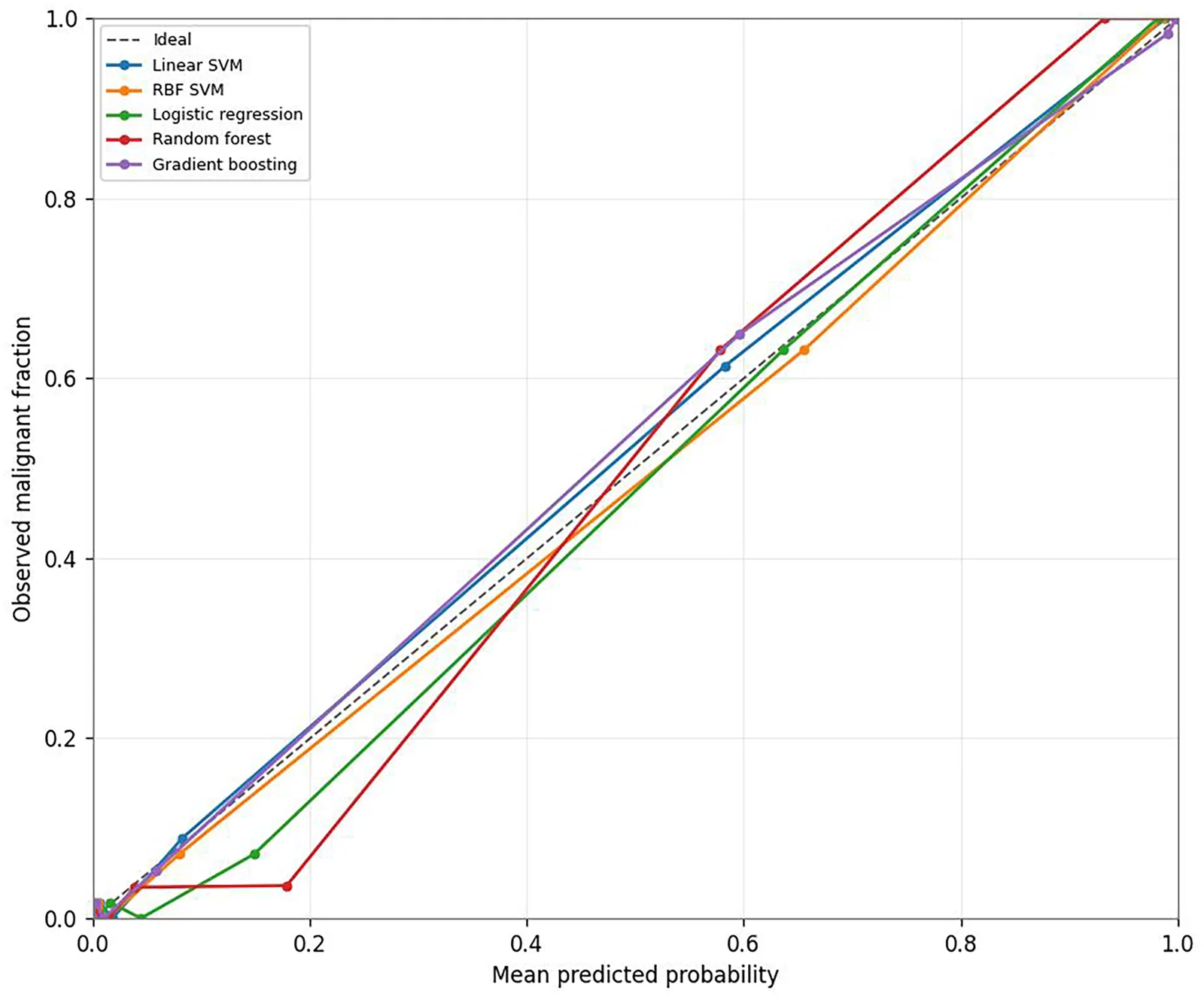
Calibration curves for the five leading diagnostic models based on outer-fold out-of-fold probabilities (n=569). Points summarize quantile-based groups of mean predicted probability and observed malignant fraction; the diagonal denotes perfect calibration.

Strictly nested Youden thresholds were 0.513, 0.406, 0.500, 0.500, and 0.560 across outer folds (median 0.500; range 0.406-0.560). Sensitivity-prioritized thresholds selected under the training sensitivity constraint were 0.112, 0.047, 0.110, 0.080, and 0.068 (median 0.080; range 0.047-0.112). Each threshold was applied only to its corresponding held-out outer fold.

**Table 4** summarizes the default operating point and aggregate held-out performance under the strictly nested Youden and sensitivity-prioritized rules. The table reports the median and range of the five training-derived thresholds, reflecting fold-specific threshold selection.

**Table 4:** Threshold-specific performance of the linear SVM using strictly nested outer-fold decisions (n=569).

Strategy or statistic	Threshold	Accuracy	Sensitivity	Specificity	Precision	F1	MCC	ROC-AUC	PR-AUC	Brier	TP/FP/FN/TN
Default threshold	0.5	0.979	0.958	0.992	0.985	0.971	0.955	—	—	—	203/3/9/354
Strictly nested Youden rule	0.500 (0.406-0.560)	0.981	0.962	0.992	0.986	0.974	0.959	0.994	0.993	0.021	204/3/8/354
95% conditional bootstrap CI	-	0.968-0.991	0.934-0.986	0.980-1.000	-	0.957-0.988	0.933-0.981	0.989-0.999	0.988-0.998	0.014-0.030	-
Strictly nested sensitivity target	0.080 (0.047-0.112)	0.912	0.991	0.866	0.814	0.894	0.831	-	-	-	210/48/2/309

Thresholds were selected from inner-fold out-of-fold predictions within each outer training partition and applied to its held-out outer fold. Threshold entries report the median (range) across five folds. The 95% intervals are stratified sample-level bootstrap percentile intervals conditional on the fixed outer-fold predictions and held-out decisions and therefore quantify conditional sampling uncertainty. ROC-AUC, PR-AUC, and Brier score are threshold-independent.

Under the strictly nested Youden rule, the linear SVM identified 204 of 212 malignant samples and correctly classified 354 of 357 benign samples, yielding 8 false negatives and 3 false positives. Aggregate held-out sensitivity was 0.962, specificity 0.992, accuracy 0.981, F1-score 0.974, and MCC 0.959. The sensitivity-prioritized rule retained 210 malignant samples, with sensitivity 0.991 (95% conditional bootstrap CI 0.976-1.000) and specificity 0.866 (0.829-0.902); false positives increased to 48.

**Figure 7** displays the aggregate confusion matrix from held-out decisions under the fold-specific Youden thresholds: 354 true negatives, 3 false positives, 8 false negatives, and 204 true positives. **Table 4** demonstrates that the sensitivity-prioritized rule reduced false negatives from 8 to 2 while increasing false positives from 3 to 48, illustrating the corresponding sensitivity-specificity trade-off. These paired outcomes provide a quantitative example for teaching threshold-dependent diagnostic reasoning. The detection of six additional malignant samples was accompanied by 45 additional false-positive classifications, illustrating the relationship between enhanced malignant-case detection and the burden of unnecessary diagnostic escalation.

**Figure 7:**
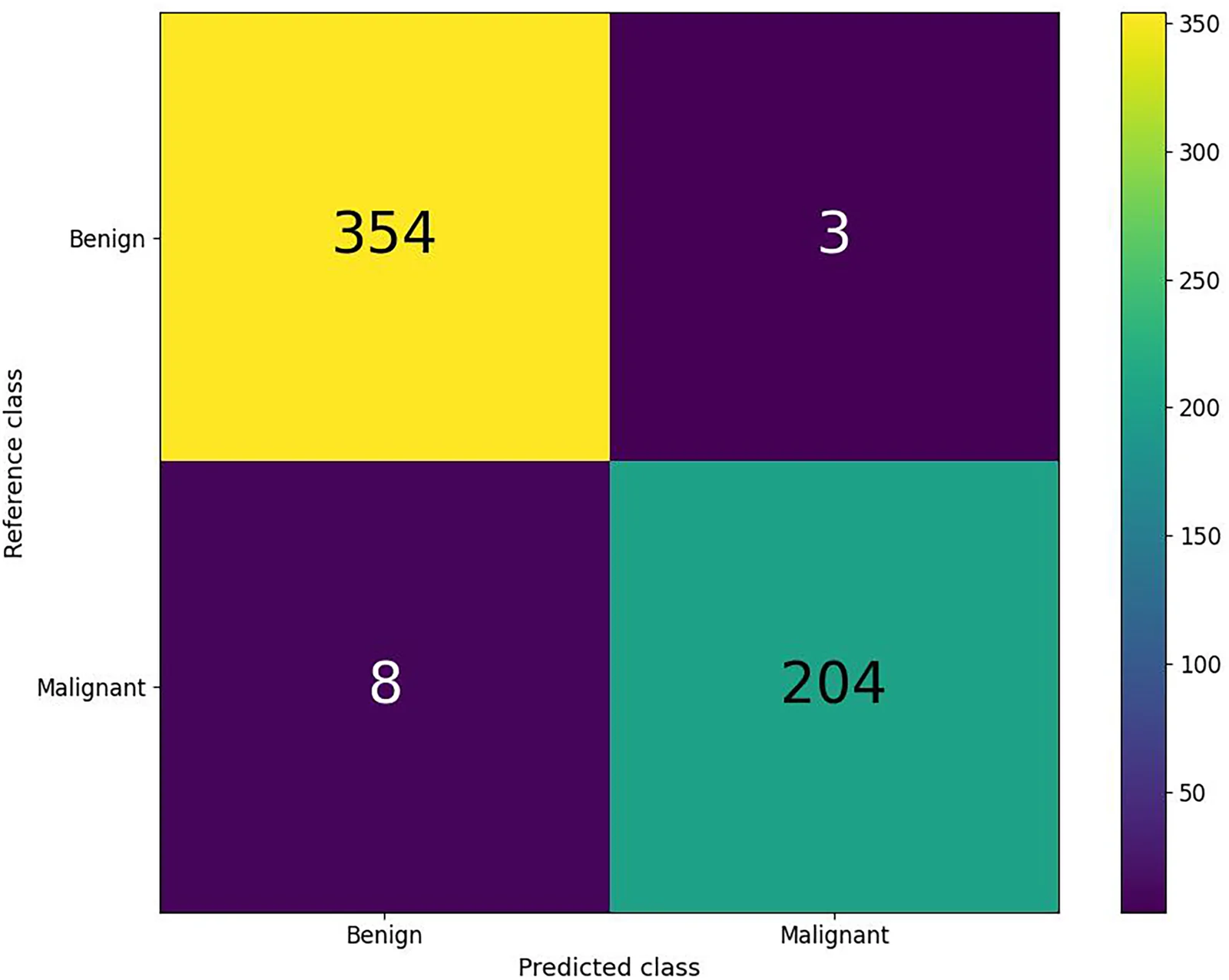
Aggregate held-out confusion matrix for the linear SVM under fold-specific Youden thresholds selected exclusively from outer-fold training data (n=569). Cells contain 354 true-negative, 3 false-positive, 8 false-negative, and 204 true-positive samples.

### 3.4 Exploratory decision-curve analysis

**Figure 8** presents exploratory decision-curve net benefit across threshold probabilities from 0.05 to 0.95. The wide range is intentionally displayed to demonstrate how net benefit changes when the relative cost assigned to false-positive escalation versus missed malignancy varies; a single fixed decision-curve threshold would represent only one such preference and would obscure this sensitivity. Interpretation remains prespecified for 0.05-0.30, representing escalation to expert cytopathology review and/or confirmatory tissue assessment. Within this range, a threshold probability of 0.05 weights a missed malignancy approximately 19 times more strongly than an unnecessary escalation, whereas 0.30 implies a ratio of approximately 2.33. The model demonstrated higher estimated net benefit than treat-all and treat-none references over most of this primary range within WDBC. Thresholds above 0.30 are displayed for completeness rather than as recommended operating points.

**Figure 8:**
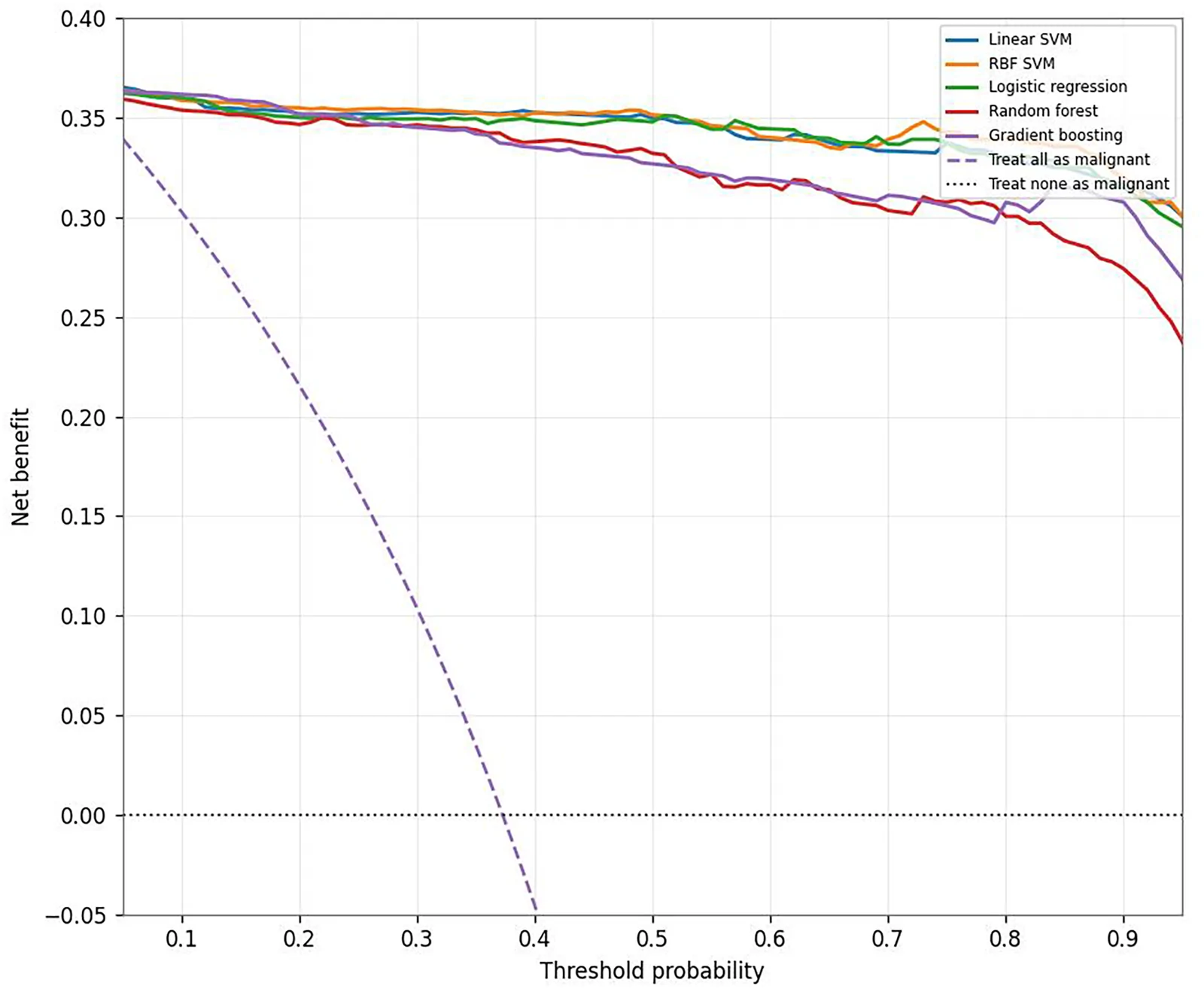
Exploratory decision-curve analysis from outer-fold out-of-fold predictions (n=569). The primary interpretive range of 0.05-0.30 represents escalation to expert review or confirmatory tissue assessment; dashed and dotted references represent escalation of all or no samples. Wider thresholds are displayed for completeness.

### 3.5 Feature importance and cytopathological plausibility

**Table 5** reports benign and malignant distributions of the top-ranked features, **Figure 9** demonstrates their permutation importance, and fold-specific standardized linear-SVM coefficients are reported below.

**Table 5:** Distributions of the top-ranked features in benign and malignant samples.

Feature	Benign mean +/- SD	Malignant mean +/- SD	Standardized mean difference
worst texture	23.52 ± 5.49	29.32 ± 5.43	1.06
radius error	0.28 ± 0.11	0.61 ± 0.35	1.27
worst symmetry	0.27 ± 0.04	0.32 ± 0.07	0.88
compactness error	0.02 ± 0.02	0.03 ± 0.02	0.62
area error	21.14 ± 8.84	72.67 ± 61.36	1.18
worst concave points	0.07 ± 0.04	0.18 ± 0.05	2.60
mean concave points	0.03 ± 0.02	0.09 ± 0.03	2.33
worst concavity	0.17 ± 0.14	0.45 ± 0.18	1.75
worst radius	13.38 ± 1.98	21.13 ± 4.28	2.32
perimeter error	2.00 ± 0.77	4.32 ± 2.57	1.23

**Figure 9:**
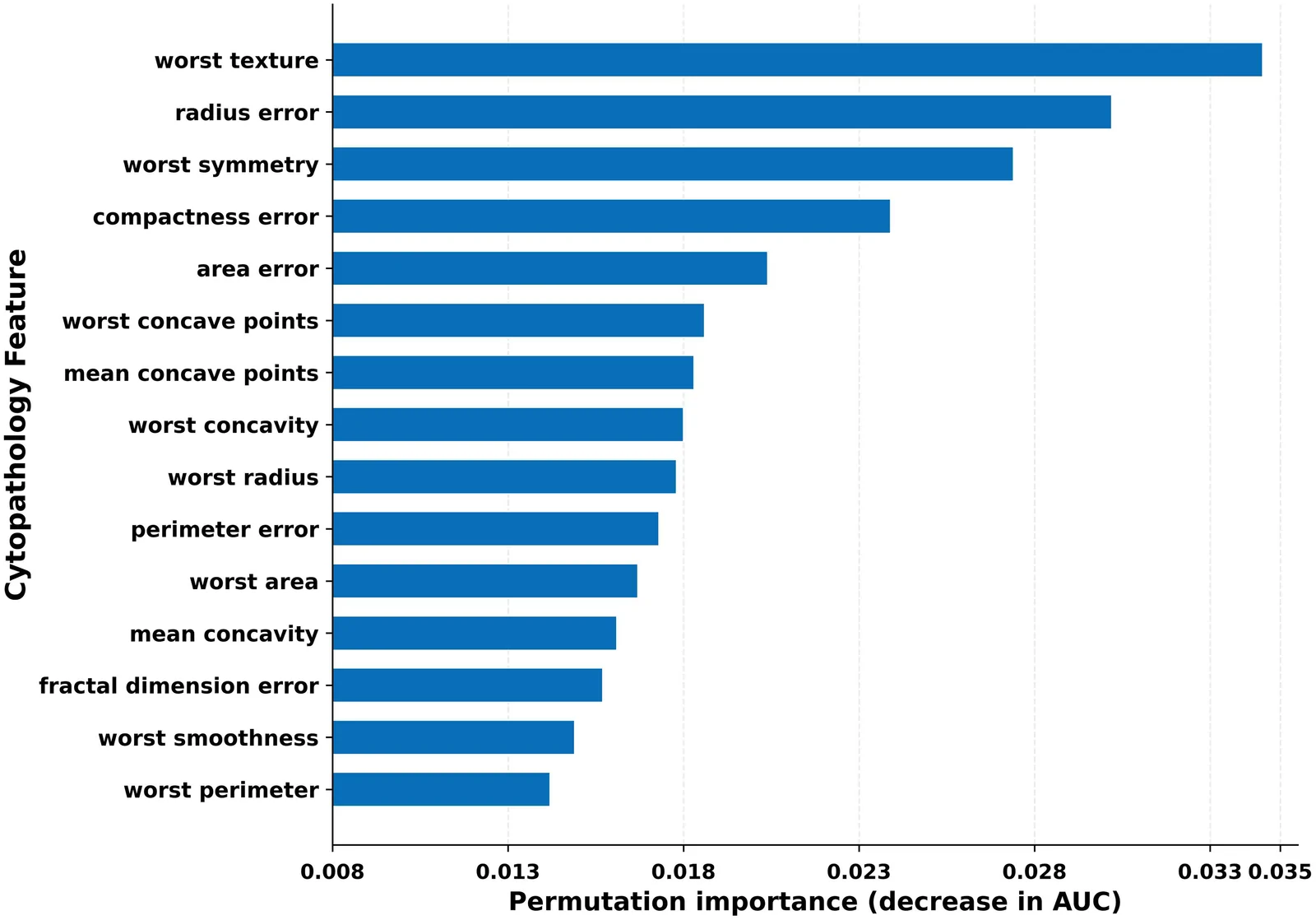
Permutation importance for the 15 leading cytopathology features. Bars display the decrease in cross-validated ROC-AUC after permuting one feature, so larger values indicate greater contribution to discrimination; correlated features may share importance.

The leading predictors were the worst texture, radius error, worst symmetry, compactness error, area error and other irregularity descriptors. **Table 5** is the class distribution, and **Figure 9** is the permutation importance. These variables are complementary measures of nuclear size, boundary irregularity, texture, symmetry and within-sample variation in malignant cytomorphology. Worst-case descriptors may be more informative because highly atypical cells can contribute significantly to malignant morphology in the measurement, and standard-error descriptors account for heterogeneity in the sampled cell population. This is in line with pleomorphism, nuclear enlargement, irregular chromatin and abnormal contours of malignant cells.

Across the five outer training folds, median standardized linear-SVM coefficients (minimum to maximum; folds with the same sign) were: worst texture 0.359 (0.194 to 0.550; 5/5), radius error 0.265 (0.134 to 0.458; 5/5), worst symmetry 0.231 (0.165 to 0.425; 5/5), compactness error -0.133 (-0.379 to -0.062; 5/5), area error 0.232 (0.122 to 0.319; 5/5), worst concave points 0.247 (0.207 to 0.380; 5/5), mean concave points 0.223 (0.161 to 0.424; 5/5), worst concavity 0.213 (0.133 to 0.443; 5/5), worst radius 0.282 (0.212 to 0.333; 5/5), and perimeter error 0.213 (0.103 to 0.380; 5/5). Positive coefficients increased the malignant decision score. The negative conditional coefficient for compactness error, despite a higher malignant-class mean, reflects multivariable adjustment among strongly correlated morphology descriptors; its interpretation is conditional on the other predictors and distinct from the positive univariable class difference.

**Table 5** provides quantitative support for pronounced class separation in nuclear size and boundary irregularity. The standardized coefficient analysis adds a conditional model perspective: nine of the ten reported features had positive coefficients in all five folds, while compactness error had a stable negative conditional coefficient because correlated predictors jointly partitioned the decision function. Accordingly, coefficient signs describe multivariable linear-model contributions, whereas standardized mean differences describe univariable class separation. The principal predictive families remained nuclear size and scale, boundary irregularity and concavity, and textural or symmetric heterogeneity. **Figure 9** demonstrates the complementary permutation-importance ranking, with larger ROC-AUC decreases indicating greater contribution to discrimination while acknowledging shared information among correlated predictors. The combined interpretation of feature distributions, correlations, permutation importance, and model coefficients connects cytopathological morphology with quantitative diagnostic reasoning. The negative conditional coefficient of compactness error, despite its positive standardized mean difference between diagnostic classes, provides a particularly informative example for teaching the distinction between univariate morphological associations and multivariable model contributions. This relationship highlights the importance of interpreting correlated nuclear descriptors within the complete diagnostic model.

### 3.6 Reduced-feature robustness analysis

**Table 6** reports performance across reduced-feature logistic-regression models, and **Figure 10** demonstrates the corresponding performance trends as the number of predictors increases.

**Table 6:** Performance of reduced-feature logistic regression models.

Number of features	AUC	Accuracy	Sensitivity	Specificity	F1	MCC
3	0.971	0.910	0.882	0.927	0.880	0.808
5	0.986	0.946	0.943	0.947	0.928	0.885
8	0.986	0.944	0.948	0.941	0.926	0.881
10	0.987	0.944	0.943	0.944	0.926	0.881
15	0.989	0.938	0.948	0.933	0.920	0.871
20	0.995	0.970	0.958	0.978	0.960	0.936
30	0.995	0.974	0.967	0.978	0.965	0.944

**Figure 10:**
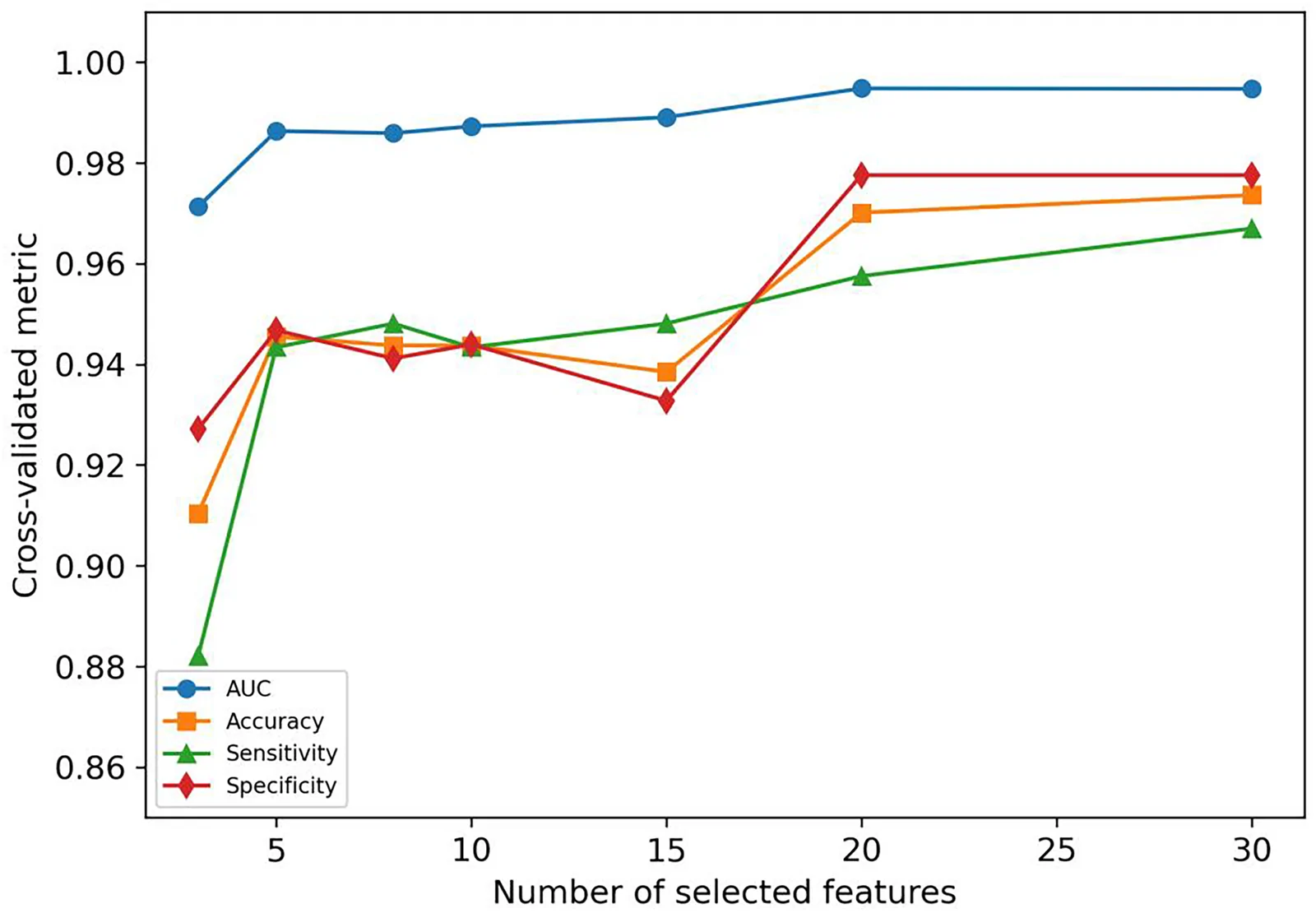
Incremental performance of reduced-feature logistic-regression models. Curves display cross-validated ROC-AUC, accuracy, sensitivity, and specificity for panels containing the top 3, 5, 8, 10, 15, 20, or 30 mutual-information-ranked features.

Reduced-feature models retained strong internal discrimination. **Table 6** reports ROC-AUC values of 0.971 with three features, 0.995 with 20 features, and 0.995 with all 30 features; **Figure 10** demonstrates the corresponding trajectory. The 20-feature specification reduced predictor count by 33.3%, representing moderate dimensionality reduction. Because WDBC variables are jointly derived from the same digitized FNA image-processing workflow, this reduction represents lower model dimensionality and simpler auditing within the shared feature-extraction process.

### 3.7 Leakage-controlled reduced-feature validation

The reduced-feature experiment demonstrated that the amount of diagnostic information gained per increase in cytopathology variables was relatively small. Leakage-controlled validation further investigated whether this pattern still occurred after ranking features only in the training folds. **Table 7** demonstrates the out-of-fold performance of logistic regression models with fold-specific mutual-information feature ranking.

**Table 7:** Leakage-controlled reduced-feature validation with fold-specific feature ranking.

Features	AUC	PR-AUC	Accuracy	Sensitivity	Specificity	F1	MCC	Brier
3	0.975	0.967	0.917	0.906	0.924	0.891	0.825	0.058
5	0.986	0.983	0.947	0.948	0.947	0.931	0.888	0.040
8	0.987	0.984	0.951	0.953	0.950	0.935	0.896	0.038
10	0.989	0.985	0.946	0.939	0.950	0.928	0.884	0.038
15	0.991	0.987	0.947	0.948	0.947	0.931	0.888	0.037
20	0.996	0.994	0.974	0.958	0.983	0.964	0.944	0.021
30	0.994	0.993	0.972	0.958	0.980	0.962	0.940	0.021

Leakage-controlled performance demonstrated an increasing trade-off between dimension reduction and predictive accuracy. A 30-feature model had an ROC-AUC of 0.994; after reducing the panel to 20 features, 33.3% of the predictors were removed, and the ROC-AUC was 0.996. The paired conditional ROC-AUC difference was 0.0012 (95% bootstrap CI -0.0004 to 0.0034), and the discrimination of the 20- and 30-feature configurations was relatively close. A stronger reduction further lowered discrimination: The 15-, 10-, 8- and 5-feature panels reduced the predictor count by 50.0%, 66.7%, 73.3% and 83.3% respectively, and reached ROC-AUCs of 0.991, 0.989, 0.987 and 0.986. For the prespecified stronger-reduction comparison, the 10-feature model differed from the 30-feature model by -0.0054 (95% bootstrap CI [-0.0117, -0.0005]), and a small but detectable loss in discrimination occurred. Threshold-dependent and probabilistic measures had the same general trend. The 20-feature and 30-feature models had the same sensitivity (0.958) and Brier score (0.021), but their accuracy was 0.974 vs 0.972, specificity 0.983 vs 0.980, F1-score 0.964 vs 0.962, and MCC 0.944 vs 0.940. Smaller panels still demonstrated some discrimination but had lower overall classification accuracy. The above results support the 20-feature configuration as a relatively reduced, performance-preserving representation.

The fold-specific top-10 panels were stable across outer folds. Mean radius, mean perimeter, mean area, area error, mean concavity, mean concave points, worst radius, worst perimeter, worst area, and worst concave points appeared in all five panels, indicating recurrent nuclear-size and boundary-irregularity signals. **Figure 11** then demonstrates how ROC-AUC and PR-AUC evolve as the fold-specific feature-panel size increases from 3 to 30.

**Figure 11:**
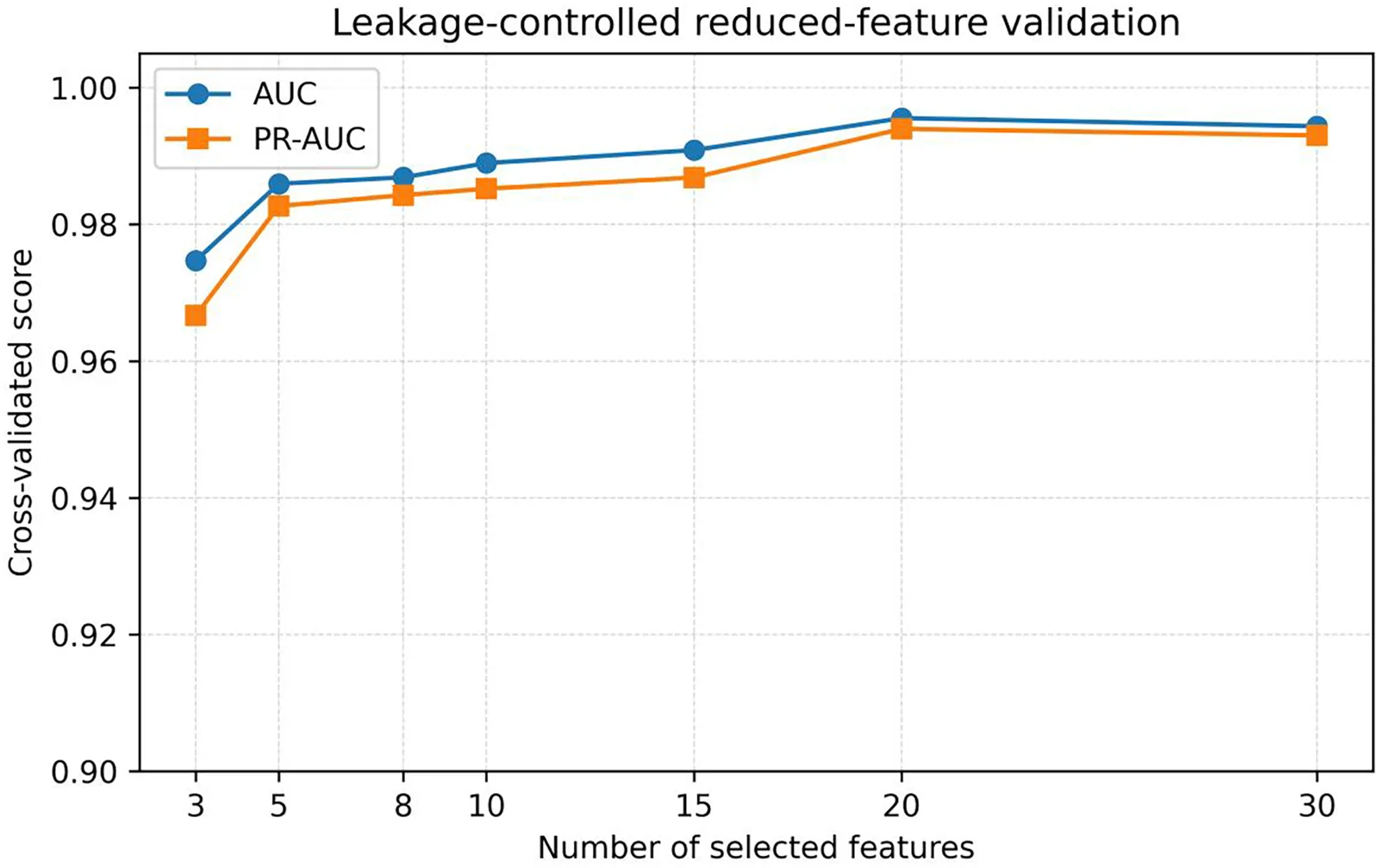
Leakage-controlled reduced-feature validation. ROC-AUC and PR-AUC were computed from outer-fold out-of-fold predictions after mutual-information ranking was repeated independently within each training fold for feature panels of size 3-30.

**Figure 11** demonstrates a rapid increase in ROC-AUC and PR-AUC from three to five features, followed by smaller gains through 15 features and near-ceiling performance around 20 features. The plateau is consistent with shared information among correlated descriptors and supports the 20-feature configuration as a moderately reduced, performance-preserving representation. The comparable discrimination of the 20-feature and 30-feature configurations offers a practical example for teaching predictor redundancy, model parsimony, and performance-preserving feature selection. The consistent recurrence of nuclear-size and boundary-irregularity descriptors across validation folds further connects the statistical stability of selected predictors with cytopathological interpretability. Together, these results illustrate how feature reduction can be evaluated through both predictive performance and morphological relevance.

### 3.8 Stability across cross-validation folds

**Figure 12** demonstrates the distribution of ROC-AUC across five outer validation folds. The leading models had high AUC values and limited dispersion among these predefined partitions, indicating internal stability within WDBC. The 95% intervals for the linear-SVM ROC-AUC and PR-AUC were 0.989-0.999 and 0.988-0.998, respectively. These bootstrap intervals condition on the fixed out-of-fold predictions and complement the fold-level assessment. Repeated-pipeline resampling would further characterize development-procedure uncertainty, while external validation is needed to determine generalization.

**Figure 12:**
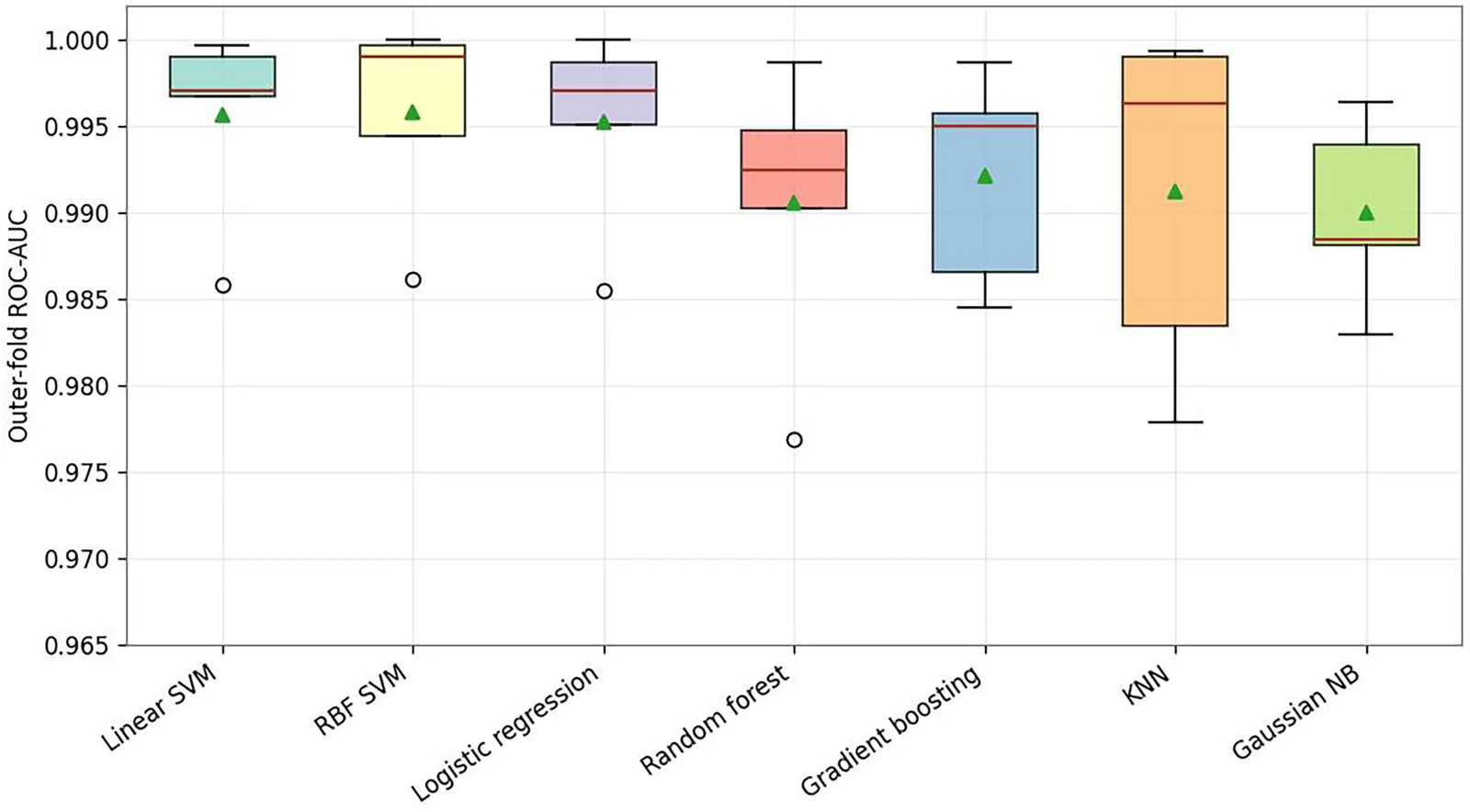
Outer-fold ROC-AUC stability across seven model families. Each box summarizes five held-out outer-fold AUC values; the center line is the median, the triangle is the mean, the box is the interquartile range, whiskers extend to 1.5 times that range, and isolated points denote values beyond the whiskers.

## 4 Discussion

Based on the above experiments, several conventional machine-learning models performed well in distinguishing between malignant and benign breast masses using quantitative cytopathology features. The RBF SVM had a slightly better numerical result in most discrimination and classification tests, but the difference between it and linear SVM and logistic regression was small. Pairwise DeLong and McNemar tests did not demonstrate any significant differences among the above leading models. Linear SVMs are relatively simple and efficient, and after standardization, the coefficients can be directly related to the impact of different shape attributes on recognition. Threshold selection also changed the diagnostic accuracy to some extent. Using fold-specific Youden’s J statistic thresholds, the linear SVM had a sensitivity of 0.962 and a specificity of 0.992; alternatively, by increasing the sensitivity threshold, it reached a sensitivity of 0.991 but lost some specificity at 0.866. Thus, the two are not equivalent in terms of overall discrimination. Exploratory decision-curve analysis also indicated a positive net benefit over a large part of the examined threshold range for escalation to expert review or confirmatory tissue examination. Probability calibration was therefore less certain than discrimination. Although the Brier score was 0.021, the calibration intercept was 0.280 and the slope was 1.284; both had relatively wide conditional confidence intervals, so the probability estimates might not be reliable for different populations with varying prevalence or case mixes.

Threshold-specific results provide a quantitative basis for teaching diagnostic decision-making. With the linear SVM, Youden-based threshold selection yielded sensitivity of 0.962 and specificity of 0.992, whereas the sensitivity-prioritized rule yielded sensitivity of 0.991 and specificity of 0.866. These paired outcomes allow the corresponding confusion matrices to be interpreted in terms of missed malignant cases and false-positive classifications. Consideration of the same predictions through calibration and exploratory decision curves extends the exercise from discrimination to probability interpretation and threshold-dependent diagnostic benefit. Together, the analyses connect statistical performance measures with decisions made under different diagnostic priorities. Most of the feature-level results were consistent with known cytological features of cancer. The types of division were nuclear size, structure, concavity, concave points, symmetry and morphological alterations. Several worst-case and variability-related indicators were relatively sensitive predictors; that is, they demonstrated significant abnormalities in the sampled cell population and correlated with nuclear enlargement, pleomorphism, irregular contours, and morphological heterogeneity. As many of the WDBC variables are highly correlated, their individual coefficients and feature-importance rankings should be interpreted in conjunction with related morphological domains, rather than as independent biological effects. Overall, the main feature groups that appeared in the malignant class included nuclear size, boundary irregularity and textural heterogeneity.

The feature-level findings offer a complementary pathway for teaching cytopathological reasoning. Measurements of nuclear size, texture, symmetry, concavity, and contour shape represent related aspects of cellular atypia and provide quantitative points of reference for malignant-versus-benign comparisons. Feature-importance results can be interpreted alongside the correlation matrix to show why highly related predictors are best considered as morphological families. This joint perspective enables the interpretation of nuclear abnormalities and model outputs within the same case-based discussion, linking familiar cytological descriptors to the numerical evidence used by a diagnostic classifier.

Progressive feature reduction creates a practical example of parsimonious model development for medical AI education. The discrimination curves across three, five, eight, ten, fifteen, twenty, and thirty selected features show how performance changes as additional predictors enter the model. The near-ceiling ROC-AUC and PR-AUC of the 20-feature analysis illustrate how a compact representation can retain substantial diagnostic information. Interpreting these patterns together with nuclear-feature correlations encourages consideration of predictor redundancy, interpretability, and the incremental value of additional measurements. Training-contained feature ranking and held-out evaluation supply a reproducible basis for examining these choices.

Reduced-feature analysis also demonstrated that the high discrimination could be maintained after moderate dimensionality reduction. The 20-feature model reduced the number of predictors by one-third and achieved an ROC-AUC close to that of the full 30-feature model, with a paired difference of 0.0012 and a 95% conditional confidence interval of [-0.0004, 0.0034]. On the other hand, reduction to 10 features demonstrated a small but noticeable decrease in discrimination. Therefore, the above analysis demonstrates that the correlated cytomorphometric variables contain considerable predictive information, and a moderately simplified feature set can retain most of the original predictive power. Training-contained preprocessing, hyperparameter selection, calibration, feature ranking, and threshold selection reduced information leakage and enabled consistent assessment of discrimination, calibration, and operating-threshold behavior.

The nested evaluation procedure also supports teaching the methodological foundations of reliable diagnostic AI. Fold-contained preprocessing, inner-fold model selection, training-derived thresholds, and outer-fold prediction assessment form a traceable sequence from original nuclear measurements to reported results. The same analytical outputs permit reconstruction of confusion matrices, comparison of probability-based and threshold-based measures, and interpretation of uncertainty intervals. Organizing these operations around one clinical classification problem links hands-on computational analysis with critical reading of model-development studies. In this way, morphological reasoning, statistical interpretation, and reproducible evaluation become connected components of a single instructional framework.

WDBC is an older, single-source dataset based on pre-extracted nuclear measurements, and it does not cover variations in specimen preparation, staining, scanning systems, segmentation methods or pathologist-dependent cell selection. Other necessary clinical data are missing, such as the patient’s age, imaging results, tumor size, histological type, receptor status, treatment and follow-up. In addition, the reported bootstrap intervals reflect sampling variability under the condition of the generated out-of-fold predictions, not the full uncertainty associated with repeating the entire model-development process. The above deficiencies limit the direct generalizability of the present results in contemporary clinical practice. Therefore, future research will examine discrimination, calibration, threshold stability and subgroup performance in independent multicenter cohorts with greater diversity in patient populations and laboratory workflows. Integrate quantitative cytomorphology with raw cytology images and breast imaging results, as well as other relevant clinical factors, to enhance the expression of diagnostic information and provide strong support for the following evaluation of clinical utility and workflow integration.

## 5 Conclusion

This paper reports a reproducible and interpretable system for malignant-versus-benign breast-mass differentiation based on WDBC nuclear morphological features for contemporaneous malignant-versus-benign diagnosis rather than future-event prediction. The top models all performed well in terms of discrimination; the RBF SVM reached a ROC-AUC of 0.995 and the linear SVM reached 0.994, and there was no significant difference between the linear SVM and the closest-performing model. Fold-specific Youden’s thresholds were derived exclusively from the corresponding outer-training partitions (median 0.500; range 0.406–0.560), and the linear SVM achieved an aggregate held-out accuracy of 0.981, sensitivity of 0.962, specificity of 0.992, F1-score of 0.974, and MCC of 0.959. Leakage-controlled reduced-feature analysis further demonstrated that a 20-feature model retained near-ceiling performance, with an ROC-AUC of 0.996 and a PR-AUC of 0.994 and thus supported a moderately reduced cytomorphometric representation that retained most of the diagnostic information. In addition, several other methods are available for assessing the general performance of the diagnostic model: nested validation, training-derived threshold selection, probability calibration, interpretable feature analysis and leakage-controlled feature reduction.

The study also establishes a case-based educational resource connecting quantitative cytopathology with medical AI evaluation. Nuclear-feature distributions and importance patterns support instruction in morphological interpretation, while the model comparisons, calibration results, and threshold-specific classifications illustrate how statistical performance informs diagnostic reasoning. The reduced-feature findings provide worked examples of predictor redundancy, model parsimony, and the interpretation of compact diagnostic representations. These linked elements form a coherent sequence from cellular measurements to classification and decision-oriented model assessment.

Further investigation will determine whether the above results are generalizable to other groups and times. Multicenter prospective cohorts should evaluate external discrimination, calibration, subgroup reliability and threshold stability under different conditions of disease prevalence, specimen preparation, staining procedures, scanning systems, segmentation methods and laboratory workflows. Combine quantitative cytomorphology with the raw cytology image and breast imaging results and other clinical factors to provide additional diagnostic information and clarify the combined functions of these data. Research on follow-up workflows will explore their clinical applicability, operational feasibility, human-AI interaction and patient-oriented outcomes before large-scale clinical application.

The open dataset and specified analytical workflow further support reproducible computational teaching. Nested cross-validation, fold-contained model development, operating-threshold comparisons, and held-out performance assessment provide a consistent set of exercises for examining the reliability and interpretability of diagnostic predictions. Integration of these quantitative tasks with the cytopathological meaning of nuclear features offers a common teaching framework for pathology, oncology, and medical data science.

## Data Availability

Publicly available datasets were analyzed in this study. This data can be found here: https://doi.org/10.24432/C5DW2B.
